# A scoping review of job search self-efficacy over the past 5 years: antecedents, mechanisms, consequences, and future directions based on SCT/SCCT

**DOI:** 10.3389/fpsyg.2025.1596847

**Published:** 2025-11-07

**Authors:** Xiaojian Zheng, Mohd Hazwan Mohd Puad, Habibah Ab. Jalil

**Affiliations:** 1 Department of Foundations of Education, Faculty of Educational Studies, Putra Malaysia University, Serdang, Selangor, Malaysia; 2 Department of Science and Technical Education, Faculty of Educational Studies, Universiti Putra Malaysia, Serdang, Selangor, Malaysia

**Keywords:** job search self-efficacy (JSSE), social cognitive career theory (SCCT), antecedents, intervention mechanisms, consequences, scoping review, social cognitive theory (SCT), job seekers

## Abstract

Against the backdrop of global labor market volatility, Job Search Self-Efficacy (JSSE) is critical for job seekers’ persistence and success. Guided by Social Cognitive Theory (SCT) and Social Cognitive Career Theory (SCCT), this scoping review synthesizes 22 empirical studies (2019–February 2025) to address three core questions: JSSE’s antecedents, intervention mechanisms, and consequences. The results showed the following: (1) antecedents concentrated on individual traits (e.g., career adaptability, emotional intelligence) and contextual support (e.g., mentoring, positive job search events); (2) intervention mechanisms were dominated by simple mediation (e.g., JSSE mediating perceived employability and job search behavior) and single moderation (e.g., extraversion moderating JSSE-success links); and (3) consequences focused almost exclusively on short-term outcomes (e.g., job search intensity, job offers). Key gaps include homogeneous samples (over-reliance on university students), methodological limitations (dominant cross-sectional designs), and understudied long-term career/emotional consequences. This review strengthens the JSSE’s theoretical connection to SCT/SCCT and provides targeted guidance for interventions while outlining directions for more inclusive and rigorous future research.

## 1 Introduction

The global labor market has faced unprecedented volatility in recent decades, driven by technological disruption (e.g., AI adoption), economic fluctuations, and shifting work models (e.g., the gig economy) (Amalia, 2023; Paslar, 2024; Yolusever, 2025). Data from the International Labour Organization (2025) show that youth unemployment reached 15.8% in 2024, whereas the World Economic Forum (2023) predicts that 23% of jobs will be restructured by 2027. In this context, job search is no longer just about “skill matching”; it depends heavily on psychological factors that support persistence and adaptation. From the unemployed during an economic crisis to university graduates, Job Search Self-Efficacy (JSSE) affects the allocation of resources to resume writing, interview rehearsals, and other phases of the job search process (Biramo et al., 2025; Liu et al., 2014a,b; Wanberg et al., 1999). JSSE, defined as an individual’s confidence in completing job search tasks, has emerged as a critical factor in shaping job seekers initiation, persistence, and success in their search efforts (Eichhorst et al., 2022; Rino et al., 2023; Wang et al., 2024).

### 1.1 SCT: the theoretical foundation

The roots of the JSSE lie in Bandura’s (1986) Social Cognitive Theory (SCT), a foundational framework for understanding human behavior that emphasizes the dynamic interplay between three core elements: personal factors (e.g., cognitive beliefs and personality), environmental factors (e.g., social support and contextual constraints), and behavior (Bandura, 1977; Foster et al., 2025; Rogers and Creed, 2011). Among SCT’s key constructs, self-efficacy (an individual’s belief in their ability to perform specific tasks) and outcome expectations (beliefs about whether task performance will lead to desired results) are identified as central drivers of behavior, shaping whether individuals initiate, persist, or adjust their actions in the face of challenges (Hackett and Byars, 1996; Hoye et al., 2019).

For example, SCT explains that an individual’s decision to pursue a goal (e.g., learning a new skill) depends on two cognitive judgments: (1) “Can I do this?” (self-efficacy) and (2) “Will doing this help me achieve what I want?” (outcome expectations). This cognitive-behavior link laid the groundwork for later extensions of SCT to specialized domains, including career development, where the complexity of vocational behavior (e.g., job search and career transition) demands a more targeted theoretical framework (Brown and Lent, 2017; Lent et al., 2000; Yu and Lee, 2015).

### 1.2 SCCT: SCT’S extension to the vocational domain

Recognizing SCT’s broad explanatory power but its need for domain-specific adaptation, Lent et al. (1991) developed the Social Cognitive Career Theory (SCCT), a theoretical model that translates SCT’s core logic into the language of career development (Brown and Lent, 2023; Lent et al., 2002). SCCT retains SCT’s focus on personal-environment-behavior interactions but refines it into a sequential, dynamic cognitive-behavior chain tailored to vocational outcomes (Damodar et al., 2024; Wang et al., 2022).

Personal Traits/Environmental Factors → Self-Efficacy/Outcome Expectations → Career Goals → Career Behavior/Career Outcomes.

Each link in this chain reflects the SCCT’s alignment with SCT while addressing the uniqueness of career-related behaviors.

(1) Personal traits and environmental factors: Building on SCT’s “personal/environmental determinants,” SCCT specifies these as career-relevant variables (e.g., personal traits such as resilience and environmental factors such as mentorship or labor market conditions) (Chuang et al., 2022). These factors do not directly shape behavior; instead, they operate through cognitive variables (self-efficacy/outcome expectations) (Ginevra et al., 2015).
(2) Self-efficacy/outcome expectations: As the “cognitive core” of SCCT (and a direct extension of SCT), these constructs mediate the effect of personal/environmental factors on goals (Arghode et al., 2021). For instance, a student with strong academic skills (personal trait) may develop high “career decision self-efficacy” (belief in their ability to choose a career path) and positive outcome expectations (belief that this choice will lead to stable employment), which together drive goal setting (Lopez et al., 1997; Ye, 2021).
(3) Career goals: SCCT frames goals as the “bridge between cognition and behavior”—goals are only formed when individuals simultaneously hold high self-efficacy (“I can achieve this goal”) and positive outcome expectations (“This goal will benefit me”) (Lent et al., 2005; Ye, 2021).
(4) Career behavior/career outcomes: Goals ultimately drive observable career behaviors (e.g., enrolling in training and applying for jobs), and the outcomes of these behaviors (e.g., securing a job and career satisfaction) are used to update self-efficacy and outcome expectations, creating a closed loop that embodies SCT’s dynamic interaction logic (Brown and Lent, 2023; Lent et al., 1991).

SCCT thus establishes a clear theoretical pathway for understanding how cognitive beliefs (especially self-efficacy) shape career development, providing the perfect framework for exploring the JSSE, a construct inherently tied to the career behavior of “job search” (Gross and Medina-DeVilliers, 2020).

### 1.3 JSSE: SCCT’S application in the job search context

JSSE emerges as a task-specific manifestation of SCCT’s “self-efficacy” construct, tailored to the unique demands of the job-search process. JSSE, defined as an individual’s perception of their ability to complete job search-related tasks (e.g., resume writing, interviewing, networking), directly aligns with SCCT’s focus on “domain-specific self-efficacy” (Brown and Lent, 2023; Lent et al., 2002; Liu et al., 2014a). Its connection to SCCT’s cognitive-behavior chain is unambiguous. Within the SCCT framework, the JSSE operates as a critical cognitive variable linking job search-relevant personal and environmental factors to job-search behavior and outcomes. For example:

(1) A job seeker with prior positive work experience (personal factor) or strong family support (environmental factor) may develop higher JSSE (e.g., “I can effectively present my skills in an interview”) and positive job search-related outcome expectations (e.g., “Interviewing well will lead to an offer”) (Fort et al., 2011).
(2) Together, these two cognitive beliefs shape specific job search goals (e.g., “I will attend three interviews this month”) (Hoye et al., 2019).
(3) Goals then drive job search behaviors (e.g., researching companies, practicing interviews), and the outcomes of these behaviors (e.g., receiving an offer, facing rejection) further update the JSSE, reinforcing SCCT’s closed-loop logic (Fort et al., 2011; Liu, Huang, et al., 2014).

Early JSSE research implicitly adopted SCCT-aligned logic. For instance, Lim et al. (2016), who proposed a social cognitive model of career self-management under SCCT (focused on process aspects of adaptive career behavior such as job searching), found that the JSSE mediates the effect of personal capabilities (e.g., job search skills) on job search intensity, a finding that directly reflects SCCT’s “personal factors → self-efficacy → behavior” pathway. Similarly, studies linking social support to JSSE (Liu et al., 2014b; Sun et al., 2013) echo the SCCT’s emphasis on environmental factors as antecedents of self-efficacy (Oh and Jun, 2023). Over time, the JSSE has evolved from a general “self-efficacy in job search” to a construct deeply embedded in SCCT’s vocational logic, making SCCT the ideal theoretical lens for systematizing JSSE research (Boudreau et al., 2001; Fort et al., 2011).

### 1.4 Rationale for examining JSSE’S antecedents, mechanisms, and consequences

While SCT and SCCT lay a solid theoretical foundation for JSSE, pioneering studies such as Kim et al. (2019) have advanced the field via a meta-analysis that quantified the correlations between JSSE and core variables (e.g., personality and social support) and verified JSSE’s association with short-term job search outcomes (e.g., application frequency and employment status) (Saks et al., 2015). However, existing research (including Kim et al., 2019) fails to fully leverage the SCCT framework to address JSSE’s three core dimensions: antecedents, intervention mechanisms, and consequences. These unaddressed gaps justify the need for a scoping review.

Regarding JSSE antecedents, Kim et al. (2019) primarily focused on quantifying the bivariate relationships between JSSE and individual factors (e.g., conscientiousness) or general environmental factors (e.g., social support). However, they did not systematically integrate the full spectrum of personal and environmental antecedents outlined in the SCCT (Taggar and Kuron, 2016). This lack of integration means that we cannot identify how multiple antecedents interact to shape the JSSE. In contrast, this scoping review systematically maps SCCT-aligned antecedents, clarifying their collective and relative impacts on JSSE.

Second, the “intervention mechanisms” linking JSSE to outcomes— a core part of SCCT’s applied value—remain underdeveloped in existing research (including Kim et al., 2019). SCCT emphasizes that self-efficacy affects behavior through modifiable cognitive and emotional processes; however, existing JSSE research has either focused on vague “mechanisms” (e.g., self-regulation) or overlooked the topic entirely (Petruzziello et al., 2020). Kim et al. (2019) further amplified this gap: while their meta-analysis confirmed correlations between social support and JSSE, it never explored the specific, actionable processes that translate these antecedents into JSSE improvements—an oversight inconsistent with SCCT’s view of self-efficacy as a malleable construct. This scoping review addresses this gap by focusing on intervention mechanisms that are critical for designing evidence-based practices for vulnerable groups (e.g., new graduates and refugees).

Third, Kim et al. (2019) limited their analysis of JSSE’s consequences to short-term, behavior-, or outcome-focused variables (e.g., interview attendance and job offers) (Vîrga and Rusu, 2018). It did not pay attention to longer-term career consequences (e.g., career adaptability and promotion potential) or emotional consequences (e.g., psychological well-being during unemployment), a limitation that restricts the understanding of the JSSE’s broader role in career development (Liu et al., 2014a; Wanberg et al., 1999, 2005). This scoping review maps consequences across short-, medium-, and long-term horizons, aligning with the SCCT’s dynamic view of lifelong career behavior (Teye-Kwadjo, 2021).

In summary, Kim et al. (2019) provided valuable quantitative insights into JSSE’s variable relationships, but their focus on correlation rather than integration (of antecedents), mechanism (intervention-focused processes), and long-term impact (of consequences) leaves key theoretical and practical gaps. By systematically synthesizing SCCT-aligned antecedents, clarifying actionable intervention mechanisms, and mapping multistage consequences, this scoping review complements Kim et al.’s (2019) findings and strengthens JSSE’s theoretical connection to SCT/SCCT. More importantly, it delivers targeted practical guidance for supporting job seekers in volatile labor markets, fulfilling the applied promise of SCCT.

### 1.5 Research problem statement

While existing research has established a preliminary theoretical prototype for JSSE based on SCCT (Lim et al., 2016), and meta-analyses such as Kim et al. (2019) have quantified correlations between JSSE and some variables, three core gaps remain: JSSE’s antecedents lack systematic integration under the SCCT framework, the specific types and operational paths of intervention mechanisms linking JSSE to outcomes are unclear, and the consequence dimension is limited to short-term indicators. To address these gaps, this study proposes the following research questions (RQs) through a scoping review:

RQ1: What categories and specific dimensions do the JSSE’s antecedent variables include?
RQ2: What specific forms do the intervention mechanisms connecting JSSE’s antecedents and consequences mainly take?
RQ3: Into which dimensions can JSSE’s consequence variables be divided, and what specific outcomes (from short-to long-term) do they cover?

## 2 Methodology

This study adopted a scoping review to systematically examine the antecedents, consequences, and mechanisms of JSSE. Adherence to the PRISMA Extension for Scoping Reviews (PRISMA-ScR) guidelines by Tricco et al. (2018) ensured the transparency and reproducibility of the review.

### 2.1 Literature retrieval and screening

#### 2.1.1 Search strategy

The search was conducted using the PsycINFO, PubMed, Web of Science, Scopus, and ProQuest Central databases, with “job search self-efficacy,” “career search self-efficacy,” and “job seeking self-efficacy” as primary keywords. To complement the evidence synthesized by Kim et al.’s (2019) meta-analysis (which covered literature up to 2018) and capture recent advancements in JSSE research, the time frame was limited to 2019–2025 (February). Supplementary searches (e.g., checking the reference lists of the included studies and articles that cite key papers) were conducted to avoid missing relevant literature.

#### 2.1.2 Selection process

Literature screening was conducted in two stages (title/abstract/keywords screening → full-text evaluation) and guided by a population-concept-context (PCC) framework—a flexible tool widely used in scoping reviews to define the study scope and ensure relevance (Arksey and O’Malley, 2005; Levac et al., 2010)—combined with explicit eligibility criteria. This ensured that only studies relevant to the “JSSE’s antecedents, intervention mechanisms, and consequences” were included.

##### 2.1.2.1 Guiding framework: PCC

To clarify the scope of the included studies, we defined the core dimensions of the PCC framework as follows:

(1) Population (Study Population)

All individuals in “job search status” or with “job search needs,” regardless of their employment history, demographic characteristics, or motivation were included. Specifically, general job seekers include undergraduates and graduates (new labor market entrants), employed job-to-job seekers, short-term unemployed (<6 months), and long-term unemployed (>6 months). Second, marginalized job seekers include refugees, ethnic minorities, individuals with disabilities, older adults (≥55 years) seeking re-employment, and low-income individuals.

Excluded: Non-job seekers (e.g., stably employed individuals without job search intentions, retirees, students without career plans) and non-human subjects research.

(2) Concept (Core Concept)

Studies focusing on at least one dimension of the JSSE (antecedents, intervention mechanisms, and consequences) were included. First, JSSE antecedents include factors that influence JSSE. Second, the JSSE intervention mechanisms include actionable processes that link the JSSE to outcomes. Third, JSSE consequences include outcomes shaped by JSSE (e.g., proximal behavioral, medium-term career, long-term career, and emotional outcomes).

Excluded: Studies on “non-job search self-efficacy” (e.g., academic self-efficacy, career decision self-efficacy without job search links) or studies that only mention JSSE but do not analyze its antecedents, mechanisms, or consequences.

(3) Context (Study Context)

Studies set in “job search-related scenarios,” regardless of the regional, cultural, or economic environment. Specifically, regional and cultural contexts include individualist and collectivist cultures and cross-cultural job searches (e.g., international migrants); economic and social contexts include routine economic environments, post-pandemic recovery periods, and gig economies; and job search stages include resume preparation, interviews, AI-assisted job search, and professional networking.

Excluded: Non-job search contexts (e.g., post-employment career development, internship selection without job search intentions).

##### 2.1.2.2 Eligibility criteria

Based on the PCC framework, additional eligibility criteria (Table 1) were defined to refine the screening process.

**Table 1:** Eligibility criteria for literature screening.

Criterion category	Inclusion criteria	Exclusion criteria
Study content	1. Aligns with PCC dimensions (relevant population, concept, context); 2. Focuses on JSSE’s antecedents, intervention mechanisms, or consequences.	1. Deviates from PCC (e.g., non-job seekers, non-JSSE concepts); 2. Only defines JSSE without empirical analysis of its core dimensions.
Study type	Empirical studies: Cross-sectional, longitudinal, experimental/quasi-experimental, case studies (with JSSE-related data);	1. Non-empirical studies: Theoretical articles, commentaries, case reports without data analysis; 2. Gray literature: Low-quality unpublished dissertations, conference abstracts (without full-text data). 3. Secondary studies: Systematic reviews, meta-analyses (to extract original study data).
Language	English (to ensure consistency in data extraction and avoid translation bias).	Non-English studies (regardless of content relevance, due to potential translation errors affecting data accuracy).
Publication time	2019–February 2025 (consistent with supplementing Kim et al.’s (2019) meta-analysis).	Before 2019 (already synthesized by prior meta-analyses).
Data completeness	Provides clear definitions of JSSE and its core dimensions (antecedents, mechanisms, and consequences), with analyzable results (e.g., correlation coefficients, thematic descriptions).	Lacks clear JSSE definitions or original data (e.g., only states “JSSE is important” without specific analysis).

##### 2.1.2.3 Screening implementation

(1) Initial screening.

After removing duplicates via Zotero, 141 records were retained for title, abstract, and keyword screening. Two researchers (ZXJ, MHMP) independently excluded records that clearly deviated from the PCC framework (e.g., “career self-efficacy of employed teachers”), resulting in 21 articles.

(2) Supplementary snowball screening.

To enhance the comprehensiveness of the literature pool, the research team conducted supplementary screening using a snowball approach by reviewing the reference lists of the 21 initially selected articles. This process identified one additional study (Mittal, 2021). The two researchers (ZXJ, MHMP) preliminarily assessed its alignment with the inclusion criteria (relevance to the JSSE’s core research direction and consistency with the PCC framework), and discrepancies were resolved through consultation with the third researcher (HAJ). All three reached a consensus that the study met the criteria, bringing the total number of candidate articles to 22.

(3) Full-text evaluation.

**Figure 1:**
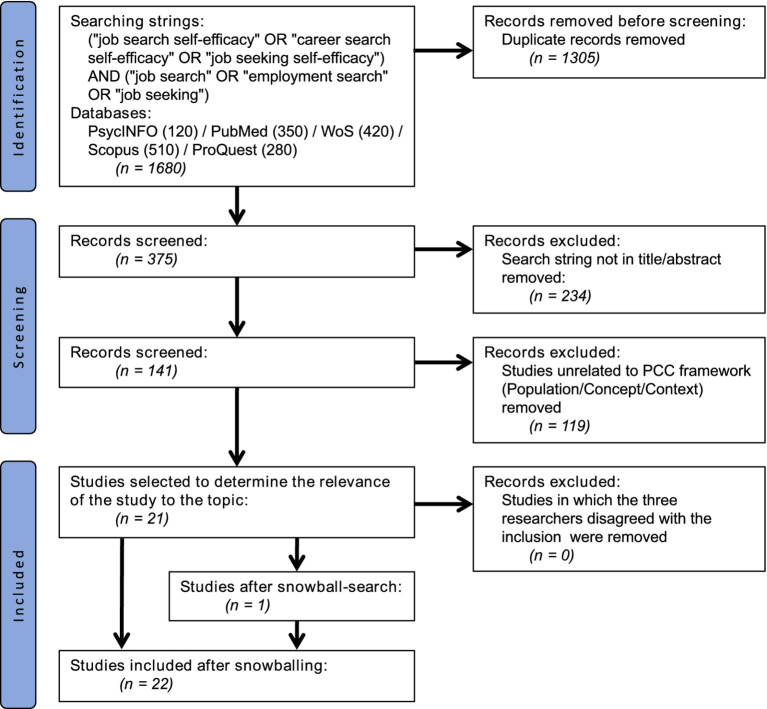
Summary of literature search, adapted from PRISMA. Figures were created by the authors.

The same two researchers (ZXJ, MHMP) assessed the relevance of the 22 full-text studies using the PCC framework and the eligibility criteria. Disagreements were resolved through discussions with the third researcher (HAJ), who served as an adjudicator. Finally, all 22 studies met the criteria and were included in the final analyses. The selection process is illustrated in Figure 1.

### 2.2 Data extraction and analysis

#### 2.2.1 Standardized data Table

Table 2 presents the study characteristics (authors, year, title), focus (which core JSSE dimension it addresses: antecedents/intervention mechanisms/consequences), key findings, and methodological details (study design, sample size, and population type). Data accuracy was verified by three researchers (ZXJ, MHMP, and HAJ) to ensure that no critical information was omitted or misrecorded.

**Table 2:** Summary of selected literature.

Title (Authors, Year)	Study design	Sample characteristics	JSSE focus dimension	Key JSSE-related findings
1. Examining the impact of a university mentorship program on student outcomes (Hamilton et al., 2019)	Mixed-methods Design (quasi-experimental pre-test/post-test with control group for quantitative data; interviews + focus groups for qualitative data)	*n* = 84; Third/Fourth-Year Undergraduate Students (and recent graduates) from a mid-sized liberal arts university in Canada (24 mentees, 62 control group participants)	- Antecedents (University Mentorship Program as a JSSE-influencing factor); - Consequences (JSSE improvement); - Contextual Focus: University-to-Work Transition	1. Quantitative: Mentees’ JSSE increased significantly over time, while the control group showed no significant JSSE change; 2. Qualitative: Mentees’ JSSE was enhanced by mentors’ career support (resume development, mock interviews, networking opportunities) and psychosocial support (anxiety reduction about post-graduation transition).
2. Job search self-efficacy as a mediator between emotional intelligence and the active job search process (Nieto-Flores et al., 2019)	Cross-sectional Study (with mediation analysis; using SPSS PROCESS macro & Bootstrap [5,000 samples]; controlling for sex, age, educational level, and unemployment duration)	*n* = 196; Unemployed Adults in Andalusia, Spain (107 men, 89 women, aged 18–59 years, M = 30.90, SD = 8.07; covering various educational levels and occupational backgrounds, including short/long-term unemployed)	- Antecedents (Emotional Intelligence as a JSSE-influencing factor); - Intervention Mechanisms (JSSE as a mediator); - Consequences (Active Job Search Process); - Contextual Focus: Unemployed Population’s Job Search	1. Emotional Intelligence positively correlates with JSSE (r = 0.60, *p* < 0.01) and active job search (r = 0.18, *p* < 0.05); 2. JSSE fully mediates the relationship between Emotional Intelligence and active job search (indirect effect 95%CI [0.022, 0.151], Sobel z = 2.82, *p* = 0.005); 3. Sociodemographic variables (sex, age, etc.) have no significant confounding effect.
3. Self-efficacy and job search success for new graduates (Petruzziello et al., 2020)	Cross-sectional Study (with mediation and moderated mediation analysis via SPSS PROCESS macro; expert interviewers’ hireability evaluation after job interview simulation; controlling for age and gender)	n = 177; Recent Graduates from a leading Italian university (66.01% female, mean age = 25.51 years, SD = 3.11); participants attended university career service job search preparation activities	- Antecedents (General Self-Efficacy [GSE] as a JSSE-influencing factor); - Intervention Mechanisms (JSSE as mediator, Extraversion as moderator); - Consequences (Job Search Success); - Contextual Focus: New Graduates’ Post-Graduation Job Search	1. GSE has no direct effect on job search success but exerts an indirect positive effect via JSSE (B = 0.18, 95%CI [0.04, 0.42]); 2. Extraversion moderates the JSSE-job search success relationship: JSSE positively predicts success for medium/high extraversion (high extraversion: B = 0.36, 95%CI [0.12, 0.79]), but not for low extraversion; 3. Job search success is measured by expert interviewers’ hireability rating (1 = not hireable, 2 = maybe, 3 = hireable) after a simulated job interview.
4. Linking mentoring and job search behavior: a moderated mediation model (Lian et al., 2021)	Two-wave Time-lagged Survey Design (with moderated mediation analysis via Bootstrap [5,000 samples]; controlling for mentee gender, mentor gender, and mentoring relationship length)	n = 596; Fourth-Year Undergraduate Students from 23 public universities in China (50.8% female, mean age = 23.2 years; 57.9% had male mentors, 65.3% maintained mentoring relationships for over 3 years)	- Antecedents (Mentoring Function as contextual support for JSSE-related processes); - Intervention Mechanisms (JSSE as moderator; Job Search Intention as mediator); - Consequences (Job Search Behavior); - Contextual Focus: Chinese Undergraduates’ School-to-Work Transition	1. Mentoring Function has a positive indirect effect on Job Search Behavior via Job Search Intention (indirect effect = 0.108, 95%CI [0.075, 0.146]); 2. JSSE positively moderates the Job Search Intention-Job Search Behavior relationship: effect is stronger for high JSSE (simple slope = 0.508, *p* < 0.001) than low JSSE (simple slope = 0.201, p < 0.001); 3. Moderated mediation is supported: indirect effect of Mentoring Function on Job Search Behavior is larger for high JSSE (0.124, 95%CI [0.086, 0.169]) than low JSSE (0.045, 95%CI [0.016, 0.085]).
5. Different starting lines, different finish times: the role of social class in the job search process (DeOrtentiis et al., 2022)	Longitudinal Study (with Structural Equation Modeling [SEM] to test JSSE mediation; Survival Analysis [Cox regression] for job acceptance rate; controlling for graduation semester, proactive personality, GPA, and gender)	n = 516; Final-Year Undergraduate Students from a large Southeastern U. S. university (69.9% White, 77.3% female, mean age = 22.4 years, SD = 2.9; all seeking full-time post-graduation employment)	- Antecedents (Social Class as a JSSE-influencing factor); - Intervention Mechanisms (JSSE as a mediator); - Consequences (Job Search Intensity, Job Acceptance Rate); - Contextual Focus: New College Graduates’ Full-Time Job Search	1. Higher social class positively predicts JSSE (*β* = 0.20, *p* = 0.04) and perceived social support (*β* = 0.28, *p* < 0.01), while negatively predicting perceived financial hardship (β = −0.50, *p* < 0.01); 2. JSSE partially mediates social class’s positive effect on job search intensity (indirect effect 95%CI [0.002,0.151], *p* < 0.05); 3. Objective social class (parental income) positively predicts job acceptance rate (hazard ratio = 1.06, *p* = 0.02), while subjective social class negatively predicts it (hazard ratio = 0.65, p = 0.02).
6. Ability-based emotional intelligence and career adaptability: role in job-search success of university students (Mittal, 2021)	Two-wave Survey Design (with hierarchical regression analysis; mediation tested via SPSS PROCESS macro [5,000 bootstrap samples]; control variables initially included but later excluded due to insignificance)	n = 729; Full-time students from a private university in India (56% male, undergraduate/postgraduate ratio ~1:1, mean age = 23.98 years, SD = 3.74)	- Antecedents (Ability-based Emotional Intelligence dimensions: Self-emotional Appraisal, Regulation of Emotion, Use of Emotion); - Intervention Mechanisms (Career Adaptability: Career Control, Career Confidence, Career Concern as mediators); - Consequences (Job Search Success); - Contextual Focus: Indian University Students’ Post-Graduation Job Search	1. Self-emotional Appraisal (B = 0.28, p < 0.05), Regulation of Emotion (B = 0.44, *p* < 0.01), Use of Emotion (B = 0.37, *p* < 0.01) positively predict job search success; Other-emotional Appraisal is insignificant; 2. Career Adaptability (Career Control/Career Confidence/Career Concern) mediates the EI-job search success relationship: full mediation for Regulation/Use of Emotion, partial mediation for Self-emotional Appraisal; 3. Career Curiosity is unrelated to all EI dimensions and does not mediate.
7. The Relationship between career adaptability and job-search self efficacy of graduates: the bifactor approach (Matijaš and Seršić, 2021)	Online Cross-sectional Study (with Confirmatory Factor Analysis [CFA] for career adaptability bifactor model; Structural Equation Modeling [SEM] for JSSE prediction; controlling for employment status)	n = 667; Master’s Graduates in Croatia (76.5% female, mean age = 25.30 years, SD = 3.28; average 2.07 months post-graduation; 49.8% employed)	- Antecedents (Career Adaptability as JSSE-influencing factor); - Contextual Focus: Croatian Graduates’ Post-Master Graduation Job Search	1. CFA confirms career adaptability fits bifactor model (general factor + 4 specific factors: concern/control/curiosity/confidence) with good fit (RMSEA = 0.06, CFI = 0.95); 2. SEM shows general career adaptability positively predicts JSSE (*β* = 0.41, 95%CI [0.28, 0.53]) and interview performance self-efficacy (IPSE, *β* = 0.39, 95%CI [0.26,0.51]); 3. Specific factors: Confidence positively predicts JSSE (β = 0.14) and IPSE (β = 0.13); Control only predicts IPSE (β = 0.17); Concern and Curiosity show no significant relationships.
8. Does feedback matter for job search self-regulation? It depends on feedback quality (Chawla et al., 2019)	Seven-week Weekly Survey (Longitudinal Design) with multilevel path analysis; temporal separation of constructs (t: feedback quality/affect; t + 1: cognitive/behavioral outcomes); controlling for gender, GPA, number of feedback organizations, and study week	n = 93; Undergraduate Business School Students (new labor market entrants) at a large southwestern U. S. university (54.8% female, mean age = 22.43 years, SD = 3.24; mean GPA = 3.27, SD = 0.34); average 4.24 weekly surveys per participant	- Antecedents (Feedback Quality, Feedback Self-Efficacy as JSSE-influencing factors); - Intervention Mechanisms (Affective Reactions, Metacognitive Strategies, Affective Rumination as mediating processes); - Contextual Focus: Weekly Job Search of New Labor Market Entrants	1. High feedback quality positively predicts positive affect (*γ* = 0.51, *p* < 0.01) and negatively predicts negative affect (γ = −0.23, p < 0.01); feedback self-efficacy weakens the negative link between feedback quality and negative affect (low efficacy: simple slope = −0.39, p < 0.01; high efficacy: non-significant); 2. Positive affect predicts subsequent metacognitive strategies (γ = 0.23, p < 0.01); negative affect predicts subsequent affective rumination (γ = 0.46, p < 0.01); 3. Metacognitive strategies positively predict weekly résumés sent (γ = 0.49, *p* < 0.05) and job search hours (γ = 1.12, *p* < 0.01); affective rumination shows no significant behavioral effects.
9. Mentoring and job search behaviors: a moderated mediation model of job search self-efficacy (Kao et al., 2021)	Two-wave Panel Design (with hierarchical regression analysis; Bootstrap [to test mediation] and simple slope test [to test moderation]; controlling for gender and age)	n = 164; Chinese College Students in their final semester (participating in a job search intervention program); 79.27% female, 20.73% male; 60.98% aged 18–25 years, 39.02% aged 26–30 years	- Antecedents (Career Mentoring, Psychosocial Mentoring as JSSE-influencing factors); - Intervention Mechanisms (JSSE as mediator; interaction of Career Mentoring × Psychosocial Mentoring); - Consequences (Job Search Behaviors); - Contextual Focus: Chinese Undergraduates’ Pre-Graduation Job Search	1. Psychosocial Mentoring positively predicts JSSE (B = 0.17, p = 0.02) and job search behaviors (B = 0.17, *p* = 0.04); Career Mentoring shows no direct effects on either; 2. JSSE fully mediates Psychosocial Mentoring’s effect on job search behaviors (indirect effect = 0.10, 95%CI [0.02,0.23]); 3. Career Mentoring × Psychosocial Mentoring interaction positively predicts JSSE (B = 0.11, *p* = 0.03): effect of Psychosocial Mentoring on JSSE is stronger for high Career Mentoring (t = 3.23, *p* < 0.01) than low (t = 0.99, *p* = 0.32); 4. JSSE positively predicts job search behaviors (B = 0.33, *p* < 0.001).
10. Specific job search self-efficacy beliefs and behaviors of unemployed ethnic minority women (Hoye et al., 2019)	Two-wave Field Study (3-month interval; hierarchical regression analysis for main effects; simple slope analysis for moderation; controlling for age, education, and number of children)	*n* = 188; Unemployed Ethnic Minority Women in the Netherlands (M = 36.28 years, SD = 9.75; 74% primary school education, 19% high school, 7% college; M = 1.80 children, SD = 1.45; 22% reemployed at Time 2; main ethnic groups: Morocco 19%, Turkey 10%, Ghana 9%)	- Antecedents (4 specific JSSE: Networking/Agency/Job Ad/Internet Self-Efficacy); - Intervention Mechanisms (Specific JSSE as moderators between job search behaviors and job offers); - Consequences (Job Search Behaviors → Job Offers); - Contextual Focus: Unemployed Ethnic Minority Women’s Job Search (Netherlands)	1. Specific JSSE predictors: Networking JSSE predicts networking behavior (*β* = 0.26, *p* = 0.005); Internet JSSE predicts Internet job search (*β* = 0.44, *p* < 0.001); Agency/Job Ad JSSE show no corresponding effects; 2. Job search behaviors → job offers: Contacting employment agencies (β = 0.33, *p* = 0.004) and looking at job ads (β = 0.27, *p* = 0.005) are positive predictors; networking/Internet search show no main effects; 3. Moderation: Networking JSSE strengthens networking-job offers (β = 0.21, p = 0.004); Job Ad JSSE strengthens job ads-job offers (β = 0.19, *p* = 0.006); 4. More children correlate with fewer job offers (β = −0.19, p = 0.04).
11. Self-efficacy dimensions and job search strategies (Fort and Puget, 2022)	Cross-sectional Study (with Confirmatory Factor Analysis [CFA] to verify scale structure; Structural Equation Modeling [SEM] for path analysis; Bootstrap to test mediation; control for measurement error via correlated error variances)	n = 120; Unemployed Adults in France (36 men, 84 women; age 19–60 years, M = 32.8, SD = 8.32; education 6–20 years, M = 13, SD = 2.80; average unemployment duration = 19 months, SD = 16.22; 67.5% faced financial pressure to find jobs quickly)	- Antecedents (Barrier Coping Efficacy, Career Decision Self-Efficacy as JSSE-influencing factors); - Intervention Mechanisms (Exploratory/Focused Strategies tested as mediators, non-significant); - Consequences (JSSE → Focused Strategy; Career Decision Self-Efficacy → Exploratory Strategy); - Contextual Focus: Unemployed Adults’ Job Search	1. CFA confirms scale validity: 3 self-efficacy dimensions (Barrier Coping/Career Decision/JSSE) and 2 job search strategies (Exploratory/Focused) fit well (CFI ≥ 0.94, RMSEA≤0.07); 2. Antecedents → JSSE: Barrier Coping Efficacy positively predicts JSSE (r = 0.686, p < 0.01) and Career Decision Self-Efficacy (r = 0.645, p < 0.01); 3. JSSE → Consequences: JSSE positively predicts Focused Strategy (*β* = 0.28, *p* < 0.01); Career Decision Self-Efficacy positively predicts Exploratory Strategy (*β* = 0.252, p < 0.01); 4. Mediation: Exploratory Strategy’s mediation between Career Decision Self-Efficacy and JSSE is non-significant (*β* = 0.07, 95%CI [−0.01,0.07]); no other significant mediations.
12. Job-search self-efficacy and reemployment willingness among older adults: roles of achievement motivation and age (Liu et al., 2021)	Cross-sectional Study (convenience sampling; PROCESS macro for moderated mediation analysis; AMOS for integrated model test; Harman’s single-factor test to control common method bias; controlling for gender, education, and monthly income)	n = 358 (effective response rate = 98.08%); Retired Older Adults in China (age 60–89 years, M = 70.93, SD = 7.94; 194 males, 171 females; 130 with primary education or lower, 42 with college education or higher)	- Intervention Mechanisms (Achievement Motivation as mediator; Age as moderator of JSSE-reemployment willingness direct path); - Consequences (JSSE → Reemployment Willingness); - Contextual Focus: Reemployment of Chinese Retired Older Adults	1. JSSE positively predicts reemployment willingness (β = 0.27, *p* < 0.001); 2. Achievement Motivation partially mediates the JSSE-reemployment willingness relationship (indirect effect = 0.07, direct effect = 0.15, p < 0.001); 3. Age moderates the direct path: JSSE predicts reemployment willingness significantly for older groups (*β* = 0.32, p < 0.001) but not for younger older groups (β = 0.12, *p* = 0.07); 4. Age negatively predicts reemployment willingness (β = −0.78, p < 0.001).
13. The quality of international mobility experiences, general self-efficacy and job search self-efficacy: A time-lagged investigation (Emirza et al., 2021)	Two-wave Time-lagged Study (average 23-month interval between Time 1/Time 2); Structural Equation Modeling (SEM) for path analysis; Bootstrap (10,000 resamples) to test mediation; EFA/CFA to verify scale structure; controlling for gender, education level, and employment status	n = 156; Turkish Graduates who participated in Erasmus Plus international mobility program (56% female, 15% participated at graduate level; 37% employed full-time; average stay abroad = 4.74 months, SD = 2.12)	- Antecedents (Quality of International Mobility Experience as JSSE-influencing factor); - Intervention Mechanisms (General Self-Efficacy as mediator between mobility quality and JSSE); - Contextual Focus: Graduates’ School-to-Work Transition (Post-International Mobility)	1. Quality of international mobility experience positively predicts General Self-Efficacy (GSE, β = 0.22, *p* = 0.02); 2. GSE positively predicts JSSE-Behavior (β = 0.68, p < 0.001) and JSSE-Outcome (β = 0.70, p < 0.001); 3. Mobility quality indirectly predicts JSSE-Behavior (β = 0.15, 95%CI [0.036,0.325]) and JSSE-Outcome (β = 0.15, 95%CI [0.038,0.331]) via GSE; 4. Scale validation: 5-factor structure for mobility quality, 3-factor structure for self-efficacies (CFI ≥ 0.91, RMSEA≤0.08) are supported.
14. The effect of perceived employability on the job search behavior of unemployed youths: the mediating role of job search self-efficacy (Biramo et al., 2025)	Mixed-methods Design (quantitative: Cross-sectional Survey with Structural Equation Modeling [SEM] for mediation; Bootstrap [1,000 samples] to test indirect effects; CFA for scale validation; qualitative: Semi-structured interviews with government staff)	n = 300 unemployed youths (213 males, 87 females) + 4 government staff (2 males, 2 females) from 6 reform towns in Wolaita, Ethiopia; youths: educated/certified, urban unemployed; staff: from Youth Association and Social Affairs Office	- Antecedents (Perceived Employability as JSSE-influencing factor); - Intervention Mechanisms (JSSE as mediator between Perceived Employability and Job Search Behavior); - Consequences (JSSE → Job Search Behavior); - Contextual Focus: Urban Unemployed Youths’ Job Search in Ethiopia	1. Quantitative: Perceived Employability positively predicts JSSE (β = 0.84, *p* < 0.05) and Job Search Behavior (β = 0.52, *p* < 0.05); JSSE positively predicts Job Search Behavior (β = 0.86, p < 0.05); 2. JSSE partially mediates Perceived Employability-Job Search Behavior (indirect effect = 0.716, 95%CI [0.497,1.104], p < 0.05); 3. Scale reliability: Perceived Employability (*α* = 0.76), JSSE (α = 0.80), Job Search Behavior (α = 0.82); 4. Qualitative: Enabling factors (skills/training) and disabling factors (discrimination, digital skill gaps) affect JSSE and job search.
15. The role of self monitoring and academic effort in students’ career adaptability and job search self-efficacy (Tolentino et al., 2019)	Cross-sectional Study (two samples; PROCESS macro for mediation/moderated mediation; Bootstrap to test indirect effects; CFA for scale validation; controlling for gender [both samples] and student year level [Sample 2])	Sample 1: n = 340; Final-year Business Students at a private Thai university (59% female, M = 22.16 years, SD = 1.21); Sample 2: n = 547; Students from a public Thai university (58% male, M = 20.64 years, SD = 1.76; 35% freshmen, 36% final-year; 10 majors including Business, Engineering)	- Antecedents (Career Adaptability as JSSE-influencing factor); - Intervention Mechanisms (Self-Monitoring as mediator; Academic Effort as moderator); - Contextual Focus: Thai University Students’ School-to-Work Transition	1. Career Adaptability positively predicts JSSE via Self-Monitoring (Sample 1: indirect effect = 0.09, 95%CI [0.04,0.15]; Sample 2: indirect effect = 0.12, 95%CI [0.06,0.25]); 2. Academic Effort strengthens Self-Monitoring-JSSE relationship: effect is stronger for high effort (B = 0.56, 95%CI [0.49,0.63]) than low effort (B = 0.39, 95%CI [0.29,0.49]); 3. Scale reliability: Career Adaptability (α ≥ 0.90), JSSE (α ≥ 0.70), Self-Monitoring (α ≥ 0.65); control variables do not alter result significance.
16. Connecting emotion regulation to career outcomes: do proactivity and job search self-efficacy mediate this link? (Urquijo et al., 2019)	Cross-sectional Study (with Structural Equation Modeling [SEM] for mediation analysis; Bootstrap [1,000 samples] to test indirect effects; Confirmatory Factor Analysis [CFA] for measurement model validation)	*n* = 399; Graduates from a private university in southern Spain (277 women, 122 men; age 22–60 years, M = 30.55, SD = 8.26; 77.7% employed, 22.3% unemployed; majors: engineering/architecture 33.1%, arts/humanities 21.6%, others including health/social sciences)	- Antecedents (Emotion Regulation as JSSE-influencing factor); - Intervention Mechanisms (JSSE as mediator; Proactivity tested as mediator, non-significant); - Consequences (JSSE → Career Outcomes: Employment Status, Salary, Contract Stability); - Contextual Focus: Spanish Graduates’ Career Development	1. Emotion Regulation positively predicts JSSE (β = 0.22, *p* < 0.001) and Career Outcomes (β = 0.16, *p* < 0.05); 2. JSSE partially mediates Emotion Regulation-Career Outcomes (indirect effect = 0.08, *p* < 0.05, 95%CI [0.011, 0.049]); 3. Proactivity shows no mediating effect (β = 0.06, ns); 4. JSSE positively predicts Career Outcomes (β = 0.30, *p* < 0.01); 5. Measurement model fits well (CFI = 0.995, RMSEA = 0.019); JSSE scale reliability α = 0.77.
17. Impact of perception reduction of employment opportunities on employment pressure of college students under COVID-19 epidemic–joint moderating effects of employment policy support and job-searching self-efficacy (Yang et al., 2022)	Cross-sectional Study (based on Stress Interaction Theory; multi-level regression for moderation/joint moderation; Harman’s single-factor test & Confirmatory Factor Analysis [CFA] for reliability/validity; controlling for gender, native place, human capital, social capital)	*n* = 810 (effective response rate = 97%); 2020–2021 Chinese College Graduates (59.6% male, 40.4% female; 69.6% rural household registration, 30.4% urban; sampled from Sichuan, Chongqing, Shanghai, etc.)	- Intervention Mechanisms (JSSE as moderator; joint moderation with Employment Policy Support); - Consequences (JSSE mitigates self/school employment pressure); - Contextual Focus: Chinese College Students’ Job Search under COVID-19	1. Perceived reduction of employment opportunities positively predicts self-pressure (β = 0.186, *p* < 0.01), school pressure (β = 0.276, *p* < 0.01), and family pressure (β = 0.148, *p* < 0.01); 2. JSSE negatively moderates perceived reduction-self-pressure (β = −0.046, *p* < 0.01): high JSSE weakens the positive effect; 3. Joint moderation: High JSSE + high Employment Policy Support minimizes perceived reduction’s impact on self/school pressure; 4. Reverse effect on family pressure: High JSSE increases family pressure (due to higher family expectations); 5. JSSE scale reliability α = 0.770; no serious common method bias (first factor explains 22.69% variance).
18. Serial multiple mediation of career adaptability and self-perceived employability in the relationship between career competencies and job search self-efficacy (Gerçek, 2024)	Cross-sectional Study (based on Career Self-Management, Career Construction Theory; Structural Equation Modeling [SEM] for model testing; Bootstrap [5,000 samples] for serial multiple mediation; Confirmatory Factor Analysis [CFA] for reliability/validity; controlling for gender and age)	*n* = 302; “Management and Organization Department” Vocational School Students from a public university in Turkey (64.9% female, 35.1% male; 58.9% aged 18–20 years, 29.8% aged 21–23 years, 11.3% aged ≥24 years)	- Antecedents (Career Competencies as JSSE-influencing factor); - Intervention Mechanisms (Career Adaptability & Self-perceived Employability as serial mediators); - Contextual Focus: Turkish Vocational School Students’ Job Search	1. Career Competencies positively predict JSSE (direct effect = 0.28, *p* < 0.05) and (total effect = 0.63, *p* < 0.05); 2. Serial mediation is supported: Career Competencies → Career Adaptability → Self-perceived Employability → JSSE (indirect effect = 0.06, *p* < 0.01, 95%CI [0.03, 0.12]); 3. All variables are positively correlated (r ≥ 0.52, *p* < 0.01); Career Adaptability predicts Self-perceived Employability (β = 0.47, *p* < 0.01), which predicts JSSE (β = 0.44, *p* < 0.05); 4. Scale reliability: JSSE (α = 0.91), Career Competencies (α = 0.97); model fit is good (CFI = 0.94, RMSEA = 0.05).
19. Implications of generational and age metastereotypes for older adults at work: the role of agency, stereotype threat, and job search self-efficacy (Weiss and Perry, 2019)	Quasi-experimental Design (one-factor between-subjects: Age Metastereotypes vs. Generational Metastereotypes; ANOVA for interaction effects; path analysis with Bootstrap [to test mediation]; controlling for gender and employment status)	*n* = 183; Adults aged 50–79 years (U.S.-based: 30% via email lists, 70% via MTurk); 55.2% male, 80.3% non-Hispanic White; 74.9% employed, 15.3% retired; split into middle-aged (50–59 years, *n* = 76) and older (60–79 years, *n* = 107)	- Antecedents (Generational/Age Metastereotypes as JSSE-influencing factors); - Intervention Mechanisms (Perceived Agency & Age-based Stereotype Threat as mediators); - Contextual Focus: Older Adults’ (60–79 years) Job Search	1. Age moderation: Effects of metastereotypes are only significant for older adults (60–79 years), not middle-aged (50–59 years); 2. Older adults: Generational metastereotypes boost Perceived Agency (M = 5.11 vs. 4.70, *p* = 0.03) and reduce Stereotype Threat (M = 3.75 vs. 4.31, *p* = 0.03) vs. age metastereotypes; 3. Mediation supported: Perceived Agency (indirect effect = 0.10, 95%CI [0.018, 0.228]) and Stereotype Threat (indirect effect = 0.12, 95%CI [0.013,0.285]) serially mediate metastereotypes-JSSE; 4. JSSE scale reliability α = 0.81; results remain robust when excluding retirees.
20. Role of perceived events in university graduates’ job search self-efficacy and success (Guan et al., 2022)	Two-wave Longitudinal Design (Time 1: 9 months pre-graduation; Time 2: 3 months pre-graduation; multiple regression + Bootstrap [5,000 samples] for mediation; controlling for baseline JSSE, job search outcomes, demographics, and self-regulation traits: career adaptability, core self-evaluation, proactive personality, approach-avoidance traits)	n = 214; Final-year Master’s Students from the School of Finance of a Beijing university, China (45.8% female, 54.2% male; mean age = 24.36 years, SD = 1.04); data collected 2018–2019 (pre-COVID-19); all seeking post-graduation employment	- Antecedents (Perceived Job Search Events: 5 positive/5 negative categories; dimensions: frequency/criticality/controllability/novelty/disruptiveness); - Intervention Mechanisms (JSSE as mediator); - Consequences (Perceived Job Search Progress, Number of Job Offers); - Contextual Focus: Pre-COVID-19 Chinese University Graduates’ School-to-Work Transition	1. Positive event criticality positively predicts T2 JSSE (β = 0.28, *p* < 0.001), while positive event novelty negatively predicts T2 JSSE (β = −0.17, *p* < 0.05); events explain 13% additional variance in T2 JSSE; 2. JSSE partially mediates: Positive criticality → T2 progress (indirect effect = 0.15, 95%CI [0.05, 0.25]) and job offers (indirect effect = 0.21, 95%CI[0.03,0.43]); positive novelty → T2 progress (indirect effect = −0.06, 95%CI[−0.11,-0.01]); 3. Direct effects: Negative event controllability → T2 progress (β = 0.22, *p* < 0.01); positive event frequency → T2 job offers (β = 0.22, *p* < 0.05); 4. JSSE scale reliability: T1 = 0.79, T2 = 0.94.
21. The impact of career adaptability and social support on job search self-efficacy: a case study in Malaysia (Al-Jubari et al., 2021)	Cross-sectional Study (Covariance Structural Equation Modeling [SEM] with maximum likelihood estimation; Confirmatory Factor Analysis [CFA] for validity; model fit assessed via CFI/RMSEA/χ^2^)	*n* = 358; Undergraduate Students from Malaysian universities (54.7% male, 45.3% female; 67.3% aged 22–25 years; 61.4% final-year students; 96.6% Malaysian nationality; CGPA mostly 3.00+, from Management & Science University, IIUM, etc.)	- Antecedents (Career Adaptability: Concern/Control/Curiosity/Confidence; Social Support); - Contextual Focus: Malaysian University Students’ Job Search	1. Career Adaptability positively predicts JSSE (β = 0.66, *p* < 0.001) and Career Outlook (β = 0.67, *p* < 0.001); 2. Social Support positively predicts JSSE (β = 0.21, *p* < 0.001) and Career Outlook (β = 0.43, *p* < 0.001); 3. JSSE scale reliability α = 0.88; model fit is good (CFI = 0.900, RMSEA = 0.075); 4. Career Adaptability’s four dimensions (all α ≥ 0.77) and Social Support (α = 0.81) show good internal consistency.
22. Increasing refugees’ work and job search self-efficacy perceptions by developing career adaptability (Morici et al., 2022)	Pre-test/Post-test Intervention Design (no control group); ESPoR career counseling intervention (2 one-hour individual interviews + 9 three-hour group meetings, ~2 months); paired t-tests for pre-post differences; linear regression for mediation; questionnaire in Italian/English/French	*n* = 233 (initial *n* = 388, 155 lost to follow-up); Refugees/asylum seekers in Italy (82.2% male, 71.6% aged 20–30 years; main origins: Pakistan 23.3%, Nigeria 22.2%; 75.6% primary/middle school education); data collected Oct 2019-Mar 2021 (pre/post-COVID-19)	- Antecedents (Career Adaptability: Concern/Control/Curiosity/Confidence); - Intervention Mechanisms (ESPoR Career Counseling Intervention → Career Adaptability → JSSE); - Consequences (JSSE improvement); - Contextual Focus: Italian Refugees’ Labor Market Integration	1. Pre-post changes: JSSE increases significantly (pre = 3.35, post = 4.04, Cohen’s d = 0.91, *p* < 0.001); Career Adaptability dimensions (Concern d = 0.88, Curiosity d = 0.86) also show large effects; 2. Career Adaptability improvement explains 82% of JSSE growth: Curiosity (β = 0.387), Concern (β = 0.264), Confidence (β = 0.204) are key predictors; 3. JSSE growth is independent of initial Career Adaptability levels; only initial Curiosity predicts WSe (Work Self-Efficacy) growth (β = 0.235, *p* = 0.002); 4. JSSE scale reliability α ≥ 0.896 (all languages); intervention includes mock interviews and labor market knowledge training.

#### 2.2.2 Systematic synthesis of JSSE’S core dimensions

To ensure that the synthesis was grounded in theoretical logic and aligned with the study’s research objectives, a deductive framework guided by theory and preset RQs was adopted to synthesize JSSE’s antecedents, intervention mechanisms, and consequences of the JSSE. This framework is based on two foundational pillars.

(1) Theoretical alignment: Derived from the SCCT, which emphasizes the sequential logic of “personal/environmental factors → self-efficacy → behavioral processes → career outcomes” (Lent et al., 1991), this framework is widely recognized for explaining the JSSE’s role in job-search behavior.
(2) Objective-driven preset dimensions: Directly mapped to the study’s core RQs, which explicitly target “JSSE’s antecedents, intervention mechanisms, and consequences” to avoid unstructured content extraction. The synthesis process was implemented in four sequential steps to ensure rigor and clarity.

##### 2.2.2.1 Preset dimension

Prior to data extraction, the operational definitions of each core dimension were clarified to ensure consistent screening.

(1) JSSE antecedents: Factors explicitly reported in studies that influence the formation or variation in JSSE levels.
(2) JSSE intervention mechanisms: Cognitive, emotional, or behavioral processes that mediate the relationship between JSSE and its outcomes.
(3) JSSE consequences: Outcomes (behavioral, career-related, or emotional) explicitly linked to JSSE, categorized as proximal (immediate behavioral responses), medium-term (short-term career results), and long-term (sustained career adaptation), based on the SCCT outcome timeline logic.

##### 2.2.2.2 Dimension-specific content extraction

Two researchers independently extracted the content from each study according to the preset dimensions. ZXJ focused on JSSE antecedents (e.g., factors shaping JSSE) and consequences (e.g., outcomes linked to JSSE, categorized by a preset timeline). MHMP focused on JSSE intervention mechanisms (e.g., processes connecting JSSE to outcomes). Both researchers referenced the PCC framework’s “Concept” dimension to ensure that the extracted content was strictly related to job search scenarios, excluding off-topic information (e.g., non-job-related self-efficacy outcomes).

##### 2.2.2.3 Cross-validation and consensus building

A third researcher (HAJ) cross-validated all the extracted content to resolve discrepancies. Disagreements (e.g., whether a factor qualified as an “antecedent” or “mechanism”) were resolved by evaluating two criteria:

(1) Explicit statements in the original study (e.g., if a study stated “Factor X affects JSSE,” X was categorized as an antecedent).
(2) Alignment with SCCT’s theoretical definitions (e.g., “mechanisms” were required to reflect dynamic processes rather than static factors).

##### 2.2.2.4 Summary of existing evidence

For each core dimension, the research team systematically summarized the evidence identified across studies, for example, by listing all antecedents reported in the literature, documenting common intervention mechanisms, and organizing consequences by preset proximal, mid-term, and long-term categories. No new themes beyond the three preset dimensions were generated, ensuring that the synthesis remained focused on the study objectives.

### 2.3 Quality control

A formal quality assessment (e.g., risk of bias) was not mandatory for scoping reviews (Tricco et al., 2018); however, objectivity was ensured. First all steps (literature screening, data extraction, dimension synthesis) were conducted independently by two researchers, with cross-checking and third-party arbitration for disagreements. Second, all screening and extraction steps (according to the PRISMA-ScR guidelines) and the rationale for excluding the120 studies were documented.

## 3 Results

### 3.1 Overview

Based on a systematic analysis of the 22 included studies, the current body of research on JSSE exhibits distinct characteristics of “three-dimensional imbalance” (across antecedents, mechanisms, and consequences) and “homogeneity in research objects and methods.” These biases not only reflect the current (2019–2025) focus of JSSE scholarship but also reveal potential gaps in theoretical exploration and practical relevance, laying a foundation for subsequent critical discussion.

#### 3.1.1 Imbalance in dimension distribution

Studies focusing on JSSE antecedents were the most prevalent, accounting for 81.8% (18/22) of the sample. These studies predominantly investigate factors that shape JSSE, with a strong emphasis on three categories: career-related resources (e.g., career adaptability; Al-Jubari et al., 2021; Gerçek, 2024), social support (e.g., mentoring programs, peer encouragement; Hamilton et al., 2019; Kao et al., 2021), and individual traits (e.g., emotional intelligence, general self-efficacy; Emirza et al., 2021; Nieto-Flores et al., 2019; Petruzziello et al., 2020). This focus is theoretically grounded in frameworks such as SCCT (Brown and Lent, 2023; Lent et al., 2002), which prioritizes identifying personal and contextual predictors of self-efficacy, an emphasis that has guided most empirical work in JSSE. Practically, antecedents are easier to operationalize and measure (e.g., using validated scales for career adaptability or emotional intelligence), making them more accessible to researchers than more abstract constructs.

Studies examining intervention mechanisms (i.e., how antecedents influence JSSE and subsequent outcomes) constitute the second-largest group, at 68.2% (15/22). However, these investigations have disproportionately concentrated on simple mediational or single moderational models. For instance, numerous studies have tested the JSSE as a mediator between antecedents (e.g., perceived employability, emotional regulation) and job search outcomes (e.g., job search behavior, employment status; Biramo et al., 2025; Urquijo et al., 2019), while others have explored single moderators such as extraversion (Petruzziello et al., 2020) or age (Liu et al., 2021) in the JSSE-outcome relationship. This preference for simple mechanisms likely stems from two factors: first, statistical tools for testing simple mediation/moderation (e.g., SPSS PROCESS macro) are widely accessible and require less complex sample designs (e.g., small to moderate cross-sectional samples); second, complex mechanisms (e.g., cross-level mediation, multi-stage serial moderation) demand larger samples, longitudinal data, and advanced analytical techniques (e.g., hierarchical linear modeling), which increase research costs and complexity.

In contrast, studies exploring the consequences of JSSE were the least common, representing only 40.9% (9/22) of the included research. Moreover, these studies are nearly exclusively limited to short-term job search outcomes, such as job search intensity (e.g., weekly job search hours; Chawla et al., 2019), the number of resumes submitted (Guan et al., 2022), or immediate employment status (e.g., number of job offers; Petruzziello et al., 2020). Long-term consequences, such as career stability (e.g., 1-year post-hiring retention), career satisfaction, and salary growth, are entirely absent from the literature. This gap can be attributed to the practical challenges of longitudinal research: tracking participants over extended periods (e.g., one to 3 years post-graduation) is time-consuming and prone to sample attrition, whereas short-term outcomes are easier to measure within a single data collection wave or short-span longitudinal design (e.g., three to 6 months). Additionally, funding constraints often discourage researchers from investing in long-term tracking studies, further reinforcing their focus on short-term consequences.

#### 3.1.2 Homogeneity in research objects

The sample populations of existing JSSE studies exhibit striking homogeneity, with university students/graduates dominating the research agenda. Of the 22 studies, 68.2% (15/22) focus on this group, including final-year undergraduate students (e.g., Hamilton et al., 2019, Canadian liberal arts university students; Kao et al., 2021, Chinese college seniors) and recent graduates (e.g., Petruzziello et al., 2020, Italian university graduates; Guan et al., 2022, Chinese master’s graduates). This overrepresentation is largely practical: university students are easily accessible through career centers or academic departments, have high response rates, and their job search processes are relatively synchronized (e.g., aligned with graduation cycles), which simplifies variable control (e.g., controlling for graduation semester; DeOrtentiis et al., 2022).

In sharp contrast, marginalized groups—such as refugees, unemployed ethnic minority women, or low-educated job seekers—are severely understudied, accounting for only 13.6% (3/22) of the sample. Examples include Morici et al. (2022), who studied refugees/asylum seekers in Italy; Hoye et al. (2019), who focused on unemployed ethnic minority women in the Netherlands; and Biramo et al. (2025), who investigated urban unemployed youth in Ethiopia. The scarcity of research on these groups reflects significant barriers to sample access: marginalized populations often require collaboration with specialized organizations (e.g., refugee shelters, community centers), may face language or cultural barriers to survey completion, and have more heterogeneous life circumstances (e.g., legal status for refugees, caregiving responsibilities for ethnic minority women) that complicate the study design.

Similarly, middle-aged and older job seekers (aged 50+) are rarely examined, representing only 9.1% (2/22) of the samples. The two exceptions are Weiss and Perry (2019), who studied adults aged 50–79 in the U.S., and Liu et al. (2021), who focused on retired older adults (60–89 years) in China. This underrepresentation may stem from the perception that the JSSE is most relevant to “first-entry” job seekers (e.g., graduates) rather than those reentering the workforce or seeking career transitions later in life. Additionally, older adults may be less likely to participate in online surveys (a common data collection method in JSSE research; e.g., Matijaš and Seršić, 2021), further reducing their inclusion.

#### 3.1.3 Homogeneity in research methods

Methodologically, existing JSSE research is heavily skewed toward cross-sectional designs, which account for 77.3% (17/22) of the studies. Cross-sectional studies (e.g., Nieto-Flores et al., 2019, Spanish unemployed adults; Fort and Puget, 2022, French unemployed adults) collect data at a single time point, making them cost-effective and efficient for exploring correlations between variables (e.g., the relationship between emotional intelligence and JSSE). However, this design cannot establish temporal order or causal relationships, a critical limitation for understanding dynamic processes such as the JSSE, which evolves over the job search period.

Longitudinal designs (including two-wave time-lagged studies) were far less common, representing only 22.7% (5/22) of the studies. Notable examples include Guan et al. (2022), who collected data 9 months and 3months before graduation, and DeOrtentiis et al. (2022), who used longitudinal data to test the JSSE’s mediating role in social class and job search outcomes. While longitudinal designs improve causal inference by capturing temporal sequences, they face significant challenges, such as sample attrition (e.g., participants dropping out between waves), increased resource requirements (e.g., repeated data collection), and the need for more sophisticated statistical analyses (e.g., handling missing data).

Quasi-experimental or experimental designs, which are best suited for testing causal relationships (e.g., the effect of an intervention on JSSE), are the rarest, at only 13.6% (3/22). These include Hamilton et al. (2019), who used a quasi-experimental pre-test/post-test design with a control group to evaluate a mentorship program, Weiss and Perry (2019), who tested the effect of metastereotype priming (age vs. generational) on JSSE, and Morici et al. (2022), who used a pre-test/post-test design to assess a career counseling intervention for refugees. The scarcity of experimental designs reflects ethical and practical constraints: assigning participants to “no-intervention” control groups may be perceived as unfair (e.g., denying mentorship to job seekers), and creating true experimental conditions (e.g., random assignment to intervention groups) is difficult in real-world job-search contexts.

In summary, the current JSSE literature is marked by imbalances in the dimension focus and homogeneity of objects and methods. These patterns not only limit the generalizability of the findings to diverse populations and contexts but also constrain the field’s ability to draw robust causal conclusions about how JSSE is shaped and how it influences long-term career outcomes.

### 3.2 Antecedents of JSSE (RQ1)

As the foundational drivers of JSSE, antecedent factors shape individuals’ confidence in executing job-search behaviors and navigating employment transitions. Based on the 22 included studies, the antecedents of JSSE can be categorized into individual trait-based and contextual support-based factors. A clear disparity emerges: research on these two categories is relatively abundant, yet it remains concentrated on specific subdomains, leaving critical gaps in understanding cultural/institutional influences, marginalized group specificity, and dynamic temporal changes.

#### 3.2.1 Well-studied domains (sufficient evidence)

##### 3.2.1.1 Individual trait-based antecedents

Individual traits—stable psychological characteristics that influence how individuals perceive and respond to job search challenges—represent the most extensively researched antecedents of JSSE, accounting for 12 of the 18 antecedent-focused studies. Two subcategories stand out: career adaptability and efficacy-related characteristics.

(1) Career Adaptability.

Career adaptability, defined as psychosocial resources that enable individuals to cope with career transitions (Savickas, 1997; Savickas and Porfeli, 2012; Zhou, 2016), was consistently identified as a robust positive predictor of JSSE across eight studies. Notably, the dimensions of concern (future-oriented planning) and confidence (self-efficacy in overcoming career obstacles) exerted the most significant effects, while control (perceived agency over career) and curiosity (exploratory tendencies) showed inconsistent or weaker associations.

For example, Al-Jubari et al. (2021) found that career adaptability collectively predicts JSSE with a standardized coefficient of *β* = 0.66 (*p* < 0.001) among Malaysian undergraduates, with post-hoc analyses revealing that concern (β = 0.32) and confidence (β = 0.38) contributed the most to this relationship. Similarly, Gerçek (2024) demonstrated in a Turkish vocational student sample that career adaptability acts as a critical mediator between career competencies and JSSE (indirect effect = 0.06, *p* < 0.01), with confidence emerging as the key mediating dimension, likely because confidence directly aligns with the “ability beliefs” core to JSSE (Bandura, 1977). Matijaš and Seršić (2021) further confirmed this pattern using a bifactor model of career adaptability: while the general adaptability factor predicts JSSE (β = 0.41, 95%CI [0.28, 0.53]), the specific confidence dimension adds incremental predictive value (β = 0.14).

The primacy of concern and confidence can be explained through SCCT: concern fosters proactive job search planning (e.g., identifying target roles and preparing materials), which builds tangible evidence of competence; confidence, in turn, translates this competence into beliefs about successful job search execution. In contrast, control and curiosity are less impactful because control focuses on “agency over career trajectory” (a broader construct than the JSSE’s task-specific confidence), and curiosity emphasizes exploration (which may not directly enhance confidence in executing job search tasks like interviewing or resume writing).

(2) Efficacy-Related Traits

Six studies highlighted the role of traits closely linked to general self-efficacy, particularly emotional intelligence (EI) and general self-efficacy (GSE), in predicting JSSE. Among these, the emotion regulation dimension of EI emerged as the most stable predictor.

Nieto-Flores et al. (2019) reported a strong positive correlation between EI and JSSE (r = 0.60, *p* < 0.01) in a sample of unemployed adults in Spain, with follow-up mediation analyses showing that EI influences active job search exclusively through JSSE (indirect effect 95%CI [0.022, 0.151]). Urquijo et al. (2019) similarly found that emotion regulation—an EI subdimension focused on managing negative affect and maintaining emotional stability—predicts JSSE (β = 0.22, *p* < 0.001) among Spanish graduates. This stability is attributed to the emotional demands of job searching: rejection, uncertainty, and competition often trigger anxiety or frustration, and individuals with strong emotion regulation can mitigate these negative effects, preserving their confidence in their job search abilities (Lent and Brown, 2013).

GSE, defined as a global belief in one’s ability to achieve goals, also contributes to JSSE indirectly. Petruzziello et al. (2020) found that GSE exerted no direct effect on job search success but operated through JSSE (B = 0.18, 95%CI [0.04, 0.42]) among Italian graduates. This aligns with the SCCT’s proposition that general efficacy beliefs are translated into domain-specific self-efficacy (e.g., JSSE) through contextual experiences—GSE provides a foundational belief in competence, which is refined into job search-specific confidence via exposure to job search tasks (e.g., career workshops, mock interviews).

##### 3.2.1.2 Contextual support-based antecedents

Contextual factors—external resources and experiences that shape JSSE— were the second major focus of antecedent research, with nine of 18 studies exploring this domain. The key subcategories include social support/mentoring and positive job search events.

(1) Social Support and Mentoring.

Seven studies confirmed that social support (from family, peers, or professionals) and mentoring programs positively enhanced JSSE, with the strongest effects observed when support combined both psychosocial (emotional, motivational) and career-specific (skill-based, informational) components.

Kao et al. (2021) distinguished between career mentoring (e.g., resume feedback and job market information) and psychosocial mentoring (e.g., anxiety reduction and encouragement) in a sample of Chinese undergraduates. While career mentoring alone showed no direct effect on JSSE, psychosocial mentoring positively predicted JSSE (B = 0.17, *p* = 0.02), and its interaction strengthened this relationship (B = 0.11, *p* = 0.03): psychosocial mentoring boosted JSSE more when paired with career mentoring (t = 3.23, *p* < 0.01) than in isolation (t = 0.99, *p* = 0.32). This “combination effect” is explained by the complementary nature of the two support types: career mentoring provides tangible skills (e.g., interview techniques) that build competence, while psychosocial mentoring reduces self-doubt, enabling individuals to translate skills into confidence (Hamilton et al., 2019).

Hamilton et al. (2019) further validated this with a mixed-methods study of Canadian university students: quantitatively, mentees’ JSSE increased significantly over time (control group showed no change); qualitatively, mentees attributed JSSE gains to both career support (e.g., networking opportunities, mock interviews) and psychosocial support (e.g., reduced post-graduation anxiety). This aligns with Bandura’s (1977) self-efficacy concept of SCT, which identifies “vicarious experiences” (observing mentors’ success) and “verbal persuasion” (mentors’ encouragement) as the key sources of self-efficacy.

(2) Positive Job Search Events.

Four studies highlighted the role of positive, context-specific events in boosting JSSE, particularly events that signal competence or provide opportunities for skill validation. These events include receiving positive feedback during interviews, securing job offers, or perceiving high employability.

Guan et al. (2022) found that the criticality (importance) of positive job search events (e.g., an interview with a desired employer) positively predicted JSSE at Time 2 (*β* = 0.28, *p* < 0.001) among Chinese master’s students, explaining 13% of the additional variance in JSSE beyond baseline levels. Critical events are impactful because they provide concrete evidence of job-search competence. For example, advancing to a final interview validates an individual’s resume quality and interview skills, reinforcing their belief in their future success.

Biramo et al. (2025) similarly showed that perceived employability (a subjective evaluation of one’s ability to secure employment) strongly predicts JSSE (β = 0.84, *p* < 0.05) among unemployed youth in Ethiopia. Perceived employability acts as a “cognitive filter” for job search experiences: individuals who perceive themselves as employable interpret ambiguous events (e.g., a recruiter’s follow-up email) as positive signals, further enhancing their JSSE. This aligns with SCCT’s emphasis on “self-reflective appraisal” as a driver of self-efficacy (Lent et al., 2000).

#### 3.2.2 Underexplored domains (evidence gaps)

While existing research provides robust evidence for individual and contextual antecedents, three critical gaps limit the generalizability and depth of understanding: cultural and institutional influences, marginalized group specificity, and dynamic temporal changes.

(1) Deep-Seated Influences of Culture and Institutions.

Only two studies (Morici et al., 2022; Weiss and Perry, 2019) acknowledge the potential role of cultural values and institutional policies in shaping JSSE antecedents, but neither explores these mechanisms in depth.

Morici et al. (2022) studied refugees in Italy—a context marked by collectivist cultural values and strict refugee employment regulations—but did not investigate how collectivism (e.g., emphasis on community support) moderates the relationship between career adaptability and JSSE. For example, in collectivist cultures, social support may be a more potent antecedent of JSSE than individual traits such as confidence, as individuals rely heavily on community validation. Similarly, Weiss and Perry (2019) examined age vs. generational metastereotypes among U. S. adults (individualist culture) but did not compare these effects to collectivist contexts, where generational identity (a collective construct) may exert a stronger influence on the JSSE than age identity (an individual construct).

Institutional policies also remain underexplored. DeOrtentiis et al. (2022) noted that social class (a proxy for access to institutional resources like career centers) predicts JSSE (*β* = 0.20, *p* = 0.04) but did not analyze how specific policies (e.g., free career counseling for low-income students) might mitigate this gap. Without understanding cultural and institutional moderators, existing antecedent models risk being “culture-bound” and unable to guide interventions in diverse contexts.

Institutional policies remain underexplored. DeOrtentiis et al. (2022) noted that social class (a proxy for access to institutional resources such as career centers) predicts JSSE (β = 0.20, p = 0.04), but did not analyze how specific policies (e.g., free career counseling for low-income students) might mitigate this gap. Without understanding cultural and institutional moderators, existing antecedent models risk being “culture-bound” and unable to guide interventions in diverse contexts.

(2) Antecedent Specificity for Marginalized Groups.

Research on JSSE antecedents is disproportionately focused on mainstream groups (e.g., university students and recent graduates), with only three studies exploring marginalized populations (refugees, unemployed ethnic minority women, and urban unemployed youth). These studies identify unique potential antecedents but fail to compare them to mainstream groups, leaving gaps in understanding “specificity.”

Morici et al. (2022) found that career adaptability predicts JSSE among refugees (β = 0.387 for curiosity, β = 0.264 for concern) but did not investigate whether “trauma recovery” (a unique experience of refugees) acts as an additional antecedent. For example, refugees with access to trauma-informed career counseling may show stronger JSSE gains than those without, yet this mechanism remains untested. Hoye et al. (2019) studied unemployed ethnic minority women in the Netherlands and found that networking JSSE predicts networking behavior (β = 0.26, *p* = 0.005) but did not compare this to majority women, leaving unclear whether networking is a more critical antecedent for minority groups (who may rely on ethnic networks for job access).

This gap has practical consequences: interventions designed for mainstream groups (e.g., focusing on individual career adaptability) may fail to address the unique needs of marginalized populations (e.g., trauma, discrimination), thereby limiting their effectiveness.

(3) Dynamic Antecedent Processes Over Time.

All 18 antecedent-focused studies adopt a static design, measuring antecedents and JSSE at one or two time points without exploring how antecedent importance changes across the job search process. For example, no study investigates whether social support is more critical for JSSE in the early job search stages (when individuals lack experience) and career adaptability becomes more important in the later stages (when individuals face rejections and need to adapt strategies).

Guan et al. (2022) collected data at two time points (9 months and 3 months pre-graduation) but analyzed the antecedents as static predictors rather than time-varying factors. This static approach ignores the dynamic nature of job search; as individuals gain experience (e.g., attend interviews, receive feedback), the antecedents driving their JSSE may shift. Without longitudinal designs that track antecedent changes, researchers cannot identify “timing-sensitive” interventions (e.g., social support early, adaptability training later), reducing the practical utility of antecedent research.

### 3.3 Intervention mechanisms of JSSE (RQ2)

Intervention mechanisms of JSSE refer to the processes through which antecedent factors (e.g., individual traits, contextual support) influence JSSE and how JSSE further shapes job search outcomes (e.g., job search behavior, employment status). These mechanisms are critical for understanding “how JSSE operates” in the job search process, as they bridge the gap between static antecedents/consequences and dynamic behavioral pathways. Based on the 22 included studies, research on JSSE intervention mechanisms is disproportionately concentrated on simple mediational and single moderational models, with limited exploration of complex, context-specific, and group-heterogeneous processes.

#### 3.3.1 Well-studied domains (sufficient evidence)

##### 3.3.1.1 Simple mediational mechanisms

Simple mediational models—where the JSSE acts as a “cognitive bridge” between antecedents and outcomes—are the most extensively investigated, accounting for 10 out of 15 mechanism-focused studies. Two subcategories dominated: JSSE as a direct mediator and single serial mediation chains involving JSSE.

(1) JSSE as a Direct Mediator.

Eight studies confirmed that the JSSE fully or partially mediated the relationship between antecedent factors and job search outcomes, reflecting its role as a core cognitive translator of resources into action. This pattern aligns with the SCCT (Brown and Lent, 2023; Lent et al., 2002), which positions self-efficacy as a key mechanism linking personal and contextual factors to career-related behavior.

For instance, Urquijo et al. (2019) demonstrated that emotion regulation (an antecedent) exerts an indirect effect on career outcomes (employment status and salary stability) exclusively through the JSSE (indirect effect = 0.08, *p* < 0.05, 95%CI [0.011, 0.049]) among Spanish graduates. Emotion regulation enhances the JSSE by helping individuals manage job search-related anxiety (e.g., coping with rejection), which, in turn, boosts their ability to secure stable employment. Similarly, Biramo et al. (2025) found that perceived employability (a subjective belief in one’s ability to find work) influences job search behavior among Ethiopian unemployed youth partially through the JSSE (indirect effect = 0.716, 95%CI [0.497, 1.104], p < 0.05). Here, JSSE serves as a “confidence filter”: individuals who perceive themselves as employable develop stronger JSSE, which motivates them to engage in more frequent job search behaviors (e.g., applying to openings and networking).

Notably, JSSE often functions as a full mediator when antecedents are intangible (e.g., emotional intelligence and general self-efficacy). Nieto-Flores et al. (2019) reported that emotional intelligence (r = 0.60, *p* < 0.01) had no direct effect on active job search; instead, its influence was entirely transmitted through the JSSE (indirect effect 95%CI [0.022, 0.151], Sobel z = 2.82, *p* = 0.005). This suggests that intangible traits require a “self-efficacy conduit” to translate into tangible behavior—individuals may possess emotional intelligence, but without the confidence to apply it to job search tasks (i.e., JSSE), they are unlikely to act on their traits.

(2) Single Serial Mediation Chains.

Two studies extended simple mediation to single serial chains, where antecedents influence JSSE through one intermediate variable, revealing a more layered mechanism. These chains highlight that JSSE is rarely shaped directly by distal antecedents; instead, it is influenced by proximal cognitive or behavioral processes.

Gerçek (2024) provided a classic example in a sample of Turkish vocational students: career competencies (e.g., skill identification, career planning) do not directly predict JSSE (direct effect = 0.28, *p* < 0.05) but exert a stronger total effect (*β* = 0.63, *p* < 0.05) through a serial chain: Career Competencies → Career Adaptability → Self-Perceived Employability → JSSE (indirect effect = 0.06, *p* < 0.01, 95%CI [0.03, 0.12]). This chain reflects a logical cognitive sequence: career competencies first build adaptability (the ability to cope with transitions), which enhances perceived employability (confidence in securing work), and finally strengthens the JSSE (confidence in job search tasks). Each step builds on the previous one, emphasizing that JSSE is a downstream outcome of cumulative cognitive development.

Another implicit serial chain was observed by Tolentino et al. (2019), who found that career adaptability influences JSSE through self-monitoring (Sample 1: indirect effect = 0.09, 95%CI [0.04, 0.15]; Sample 2: indirect effect = 0.12, 95%CI [0.06, 0.25]) among Thai university students. Self-monitoring, or awareness of one’s strengths and weaknesses, acts as a bridge: adaptable individuals are more likely to reflect on their job search performance, which refines their JSSE by aligning their confidence with their actual capabilities.

##### 3.3.1.2 Single moderational mechanisms

Seven studies explored single moderational models, where a third variable (individual trait or contextual factor) altered the strength or direction of the relationships involving the JSSE. These models address “when JSSE is most impactful” or “when antecedents most strongly shape JSSE,” providing nuance to the one-size-fits-all assumptions of simple mediation analyses.

(1) Individual Trait Moderators.

Five studies identified individual traits as key moderators of JSSE-outcome relationships, with extraversion and achievement motivation emerging as the most consistent moderators. These traits influence how individuals translate their JSSE into action by shaping their willingness to engage with job search opportunities.

Petruzziello et al. (2020) found that extraversion moderates the JSSE-job search success relationship among Italian graduates: JSSE positively predicts hireability (as rated by experts) for medium (*β* = 0.22, p < 0.05) and high extraversion (β = 0.36, 95%CI [0.12, 0.79], p < 0.01) but has no effect on low extraversion (β = 0.08, ns). Extraversion enhances the JSSE-success link because extroverted individuals are more likely to act on their JSSE; they engage in more networking, communicate more effectively in interviews, and proactively pursue openings. In contrast, introverted individuals may possess a high JSSE but lack the social engagement needed to translate it into success.

Liu et al. (2021) similarly showed that achievement motivation strengthens the direct effect of JSSE on reemployment willingness among Chinese retired older adults (indirect effect = 0.07, *p* < 0.001). Achievement-motivated individuals view JSSE as a signal to pursue challenging reemployment goals (e.g., full-time rather than part-time work), whereas those with low achievement motivation may not act on their JSSE, even if they possess it.

(2) Contextual moderators.

Two studies highlighted contextual factors as moderators of JSSE-related processes, emphasizing that environmental resources can amplify or dampen the impact of JSSE or its antecedents. The most prominent contextual moderators are employment policy support and mentoring type.

Yang et al. (2022) investigated the joint moderation of JSSE and employment policy support among Chinese college graduates during the COVID-19 pandemic. They found that JSSE negatively moderated the relationship between “perceived reduction in employment opportunities” and self-reported job search pressure (β = −0.046, *p* < 0.01)—but this buffering effect was strongest when paired with high employment policy support (e.g., government job fairs, subsidies). High policy support provides tangible resources (e.g., access to openings) that validate the JSSE, making individuals more confident in their ability to cope with limited opportunities. Without policy support, the JSSE alone is insufficient to reduce pressure, as individuals lack the practical means to act on their confidence.

Kao et al. (2021) further showed that mentoring type (career vs. psychosocial) moderates the relationship between psychosocial mentoring and JSSE among Chinese undergraduate students. Psychosocial mentoring (e.g., emotional support) positively predicted JSSE (B = 0.17, *p* = 0.02), but this effect was stronger when combined with career mentoring (e.g., resume feedback; interaction effect B = 0.11, *p* = 0.03). Career mentoring provides concrete evidence of competence (e.g., improving a resume), which reinforces the emotional support of psychosocial mentoring; together, they create a “confidence loop” that strengthens JSSE more than either alone.

#### 3.3.2 Underexplored domains (evidence gaps)

While simple mediation and moderation models provide foundational insights, three critical gaps limit the practical and theoretical utility of JSSE intervention mechanisms: neglect of complex mechanisms, lack of granularity in intervention component analysis, and absence of group heterogeneity testing.

##### 3.3.2.1 Exploration of complex mechanisms

Only one study (Lian et al., 2021) investigated moderated mediation, a more complex mechanism whichere a moderator alters the strength of a mediational pathway, with no research exploring multistage serial moderated mediation or cross-level mechanisms. This gap oversimplifies the dynamic nature of job search processes, in which multiple factors interact to shape the JSSE.

Lian et al. (2021) demonstrated moderated mediation in a sample of Chinese undergraduates: mentoring function influences job search behavior through job search intention, and this indirect effect is stronger for individuals with high JSSE (indirect effect = 0.124, 95%CI [0.086, 0.169]) than low JSSE (indirect effect = 0.045, 95%CI [0.016, 0.085]). However, no study has extended this to more realistic scenarios, such as “serial moderated mediation” (e.g., JSSE moderates the first step of a serial chain: Career Adaptability → Self-Perceived Employability → JSSE → Job Search Behavior) or “cross-level mechanisms” (e.g., organizational age diversity climate moderates individual-level JSSE’s effect on job search behavior).

Cross-level mechanisms are particularly critical in organizational contexts, where group-level factors (e.g., team support and company hiring policies) can shape how individuals translate JSSE into action. For example, an organization with a supportive job search culture (e.g., allowing employees to use work time for job search) may amplify the effect of JSSE on job search intensity, but this remains to be tested. Without exploring complex mechanisms, researchers cannot capture the “real-world complexity” of the JSSE’s operation.

##### 3.3.2.2 Specific pathways of intervention measures

Only three studies (Morici et al., 2022; Hamilton et al., 2019; Kao et al., 2021) mention formal interventions (e.g., career counseling, mentoring programs) as influencing JSSE, but none disaggregate the key components of these interventions (e.g., mock interviews, resume workshops) or their unique effects on JSSE. This gap limits the practical utility of the research, as practitioners cannot determine which intervention elements are most effective for boosting the JSSE.

Morici et al. (2022) evaluated an ESPoR career counseling intervention for refugees, which included mock interviews, labor market knowledge training, and individual career planning. They found that the intervention increased JSSE (pre = 3.35, post = 4.04, Cohen’s d = 0.91, *p* < 0.001), but did not test whether mock interviews (which build interview-specific confidence) or labor market training (which reduces uncertainty) drove this effect. Similarly, Hamilton et al. (2019) found that university mentorship boosted JSSE but did not isolate whether resume development (skill-focused) or anxiety reduction (psychosocial) was more impactful.

This lack of granularity is problematic because different intervention components may target distinct aspects of JSSE: mock interviews may enhance “interview performance self-efficacy” (a subdimension of JSSE), while resume workshops may boost “application-specific self-efficacy.” Without isolating these components, interventions risk being resource-inefficient, as practitioners may include unnecessary elements or omit high-impact ones.

##### 3.3.2.3 Group heterogeneity in mechanisms

No study has compared JSSE mechanisms across different population groups (e.g., students vs. unemployed adults, collectivist vs. individualist cultures), assuming that the mechanisms are universal. This assumption is questionable, as group-specific experiences (e.g., unemployment duration and cultural values) likely shape how the JSSE operates.

For example, JSSE may mediate the relationship between social support and job search behavior differently for students and unemployed adults: students may rely on peer support to build JSSE, whereas unemployed adults may depend more on family or professional support. Similarly, in collectivist cultures (e.g., China, Malaysia), JSSE may be more strongly moderated by “family approval” (a contextual factor), whereas in individualist cultures (e.g., the U. S., Spain), it may be moderated by “personal autonomy.”

Hoye et al. (2019) studied unemployed ethnic minority women in the Netherlands and found that networking JSSE predicts networking behavior (*β* = 0.26, *p* = 0.005)—but they did not compare this to majority women, leaving unclear whether networking is a more critical mechanism for minority groups (who may rely on ethnic networks for job access). Without testing group heterogeneity, mechanism findings risk being “one-size-fits-all” and may fail to guide interventions for marginalized or culturally distinct populations.

### 3.4 Consequences of JSSE (RQ3)

The consequences of JSSE refer to the tangible and intangible outcomes resulting from individuals’ confidence in executing job search tasks. These outcomes are critical for evaluating the practical value of the JSSE—whether it merely shapes subjective perceptions or drives meaningful changes in job search behavior and long-term career trajectories. Based on the 22 included studies, research on JSSE consequences is highly skewed toward short-term, job search-specific outcomes, with minimal exploration of long-term career impacts, non-employment-related spillover effects, or contextual boundary conditions that may alter the JSSE’s influence.

#### 3.4.1 Well-studied domains (sufficient evidence)

Nearly all (seven out of nine) consequence-focused studies concentrate on short-term job search outcomes, which are defined as outcomes observable within weeks to months of job search initiation. These outcomes fall into two overlapping categories: job search behavior and immediate employment status. This focus reflects the practicality of measuring short-term outcomes (e.g., via weekly surveys or post-graduation follow-ups) and aligns with the primary goal of most job search studies: supporting individuals in securing employment quickly.

#### 3.4.2 Job search behavior

Five studies consistently demonstrate that JSSE positively predicts job search behavior—the effort, intensity, and strategic choices individuals invest in their job search. These behaviors are considered “proximal consequences” of JSSE, as they directly reflect how efficacy beliefs translate into action (Bandura, 1986).

Fort and Puget (2022) found that the JSSE positively predicted the adoption of focused job search strategies (*β* = 0.28, *p* < 0.01) among unemployed adults in France. Focused strategies, such as targeting specific industries or roles, are more efficient than exploratory strategies, suggesting that individuals with high JSSE prioritize quality over quantity in their job search. This aligns with self-regulation theory: individuals with strong JSSE are more likely to select strategies they believe will succeed rather than wasting effort on unfocused exploration (Liu et al., 2014b).

Chawla et al. (2019) further validated this with a seven-week longitudinal study of U. S. business students: JSSE indirectly predicted weekly job search hours (*γ* = 1.12, *p* < 0.01) and the number of resumes sent (γ = 0.49, *p* < 0.05) through metacognitive strategies (e.g., planning daily job search tasks). Individuals with high JSSE are more likely to engage in proactive self-regulation (e.g., setting daily goals for applications), which increases their overall efforts.

Additional support comes from Biramo et al. (2025), who reported a strong positive relationship between JSSE and job search behavior (β = 0.86, *p* < 0.05) among urban unemployed youth in Ethiopia; Lian et al. (2021), who showed that JSSE strengthens the link between job search intention and behavior (high JSSE: simple slope = 0.508, *p* < 0.001; low JSSE: simple slope = 0.201, *p* < 0.001) for Chinese undergraduates; and Kao et al. (2021), who found that JSSE directly predicts job search behaviors (B = 0.33, *p* < 0.001) in a sample of Chinese college seniors. Collectively, these studies confirm that the JSSE acts as a “behavioral driver”: individuals who believe they can succeed in job search tasks are more willing to invest time, effort, and strategic thought into their search.

##### 3.4.2.1 Immediate employment status

Four studies extended the behavioral consequences of JSSE to short-term employment outcomes, such as the number of job offers, hireability ratings, and employment status. These outcomes are considered “distal consequences” of the JSSE, as they depend on both behavior and external factors (e.g., employer decisions); however, the JSSE still plays a critical role in increasing the likelihood of success.

Guan et al. (2022) found that JSSE partially mediated the relationship between positive job search events (e.g., critical interviews) and the number of job offers (indirect effect = 0.21, 95%CI [0.03, 0.43], *p* < 0.05) among Chinese master’s students. High JSSE individuals are more likely to persist through the interview process (e.g., following up with recruiters, preparing for multiple rounds), which increases their chances of receiving job offers.

Petruzziello et al. (2020) provided experimental evidence that the JSSE positively predicts expert-rated hireability (high extraversion: B = 0.36, 95%CI [0.12, 0.79], *p* < 0.01) among Italian graduates after a simulated job interview. The JSSE enhances interview performance by reducing anxiety and increasing confidence, which recruiters perceive as competence.

Urquijo et al. (2019) and DeOrtentiis et al. (2022) further support this pattern: Urquijo et al. (2019) found JSSE predicts stable employment status (*β* = 0.30, *p* < 0.01) among Spanish graduates, while DeOrtentiis et al. (2022) reported that JSSE partially mediates the effect of social class on job search intensity (indirect effect 95%CI [0.002, 0.151], *p* < 0.05), which in turn increases the likelihood of securing full-time employment.

The consistency of these findings can be explained by the SCCT (Lent et al., 1991): JSSE increases persistence in the face of rejection (a common job search barrier), leading to more applications, better interview performance, and ultimately, higher short-term employment success.

#### 3.4.3 Underexplored domains (evidence gaps)

While short-term consequences are well documented, three gaps limit our understanding of the JSSE’s full impact: the absence of long-term career outcomes, the neglect of non-employment-related spillover effects, and the lack of research on contextual boundary conditions.

##### 3.4.3.1 Long-term career consequences

No study in the sample explored the long-term career impacts of JSSE, such as job retention (e.g., 1-year post-hiring turnover), career satisfaction, or salary growth. This gap is significant because JSSE’s value should not be limited to “securing a job” but also to “sustaining career success.”

For example, a graduate with a high JSSE may secure a job quickly, but if the job is misaligned with their skills or values (due to hasty decision-making), they may experience high turnover or low satisfaction. Conversely, JSSE could enhance long-term career adaptability; individuals who developed JSSE during their initial job search may be more confident in navigating future career transitions (e.g., promotions and industry changes). Without longitudinal studies tracking outcomes beyond 6–12 months, we cannot determine whether JSSE contributes to “career success” or merely “short-term employment.”

The lack of long-term research likely stems from practical challenges, as tracking participants for multiple years is time-consuming and costly, and funding for such studies is limited. However, this gap reduces the utility of JSSE research for career counselors and policymakers, who need evidence that JSSE-building interventions yield sustained benefits.

##### 3.4.3.2 Non-employment-related spillover effects

Only one study (Liu et al., 2021) touches on non-employment consequences, specifically the relationship between JSSE and reemployment willingness (β = 0.27, *p* < 0.001) among Chinese retired older adults. No study has explored the JSSE’s spillover effects on mental health (e.g., job search anxiety, depression) or overall life satisfaction, despite job search being a stressful process with significant psychological impacts.

A high JSSE could act as a “buffer” against job search-related anxiety: individuals who believe they can succeed are less likely to feel overwhelmed by rejection or uncertainty. Conversely, low JSSE may exacerbate anxiety, creating a “negative cycle” in which anxiety reduces job search effort, further lowering JSSE. For marginalized groups (e.g., refugees and unemployed ethnic minorities) who face additional job search barriers, this cycle can be particularly damaging.

The neglect of spillover effects reflects a narrow focus on “employment as the sole outcome” in job-search research. However, mental health and life satisfaction are critical for holistic well-being; an intervention that boosts JSSE to reduce anxiety may be valuable even if it does not immediately increase employment rates.

##### 3.4.3.3 Boundary conditions of JSSE-consequence relationships

No study has examined the contextual factors that may strengthen or weaken the relationship between JSSE and its consequences, such as economic conditions (recession vs. prosperity) or job type (manual vs. knowledge work). This omission assumes that the JSSE operates uniformly across contexts, which is unlikely.

For example, during an economic recession (e.g., COVID-19), high JSSE may have a weaker effect on employment outcomes because job openings are scarce; even confident job seekers may struggle to secure offers. Yang et al. (2022) noted that the JSSE mitigated job search pressure during COVID-19 but did not test whether the JSSE’s effect on employment was reduced compared to pre-pandemic periods.

Similarly, JSSE may be more impactful for knowledge work (e.g., professional roles) than manual labor (e.g., construction), as knowledge work often requires interpersonal skills (e.g., interviewing, networking), where confidence (JSSE) translates to performance. By contrast, manual labor may rely more on physical skills than self-efficacy. Without testing these boundary conditions, we cannot tailor JSSE-building interventions to specific contexts; for example, we cannot emphasize JSSE more for professional job seekers during economic booms.

In summary, the JSSE’s consequences are well documented for short-term job search behavior and employment status, but critical gaps remain in long-term career impacts, non-employment spillover effects, and contextual boundary conditions. Addressing these gaps is essential to fully understand JSSE’s role in supporting not just “getting a job” but also “building a sustainable, satisfying career.”

### 3.5 Contradictory findings

While most JSSE studies converge on core relationships (e.g., positive links between social support and JSSE), several contradictory findings have emerged across studies. These contradictions are not trivial; they reflect unaddressed heterogeneity in sample characteristics, measurement approaches, or contextual factors, which may undermine the generalizability of JSSE theories and the effectiveness of practice interventions. Table 3 synthesizes the key contradictory relationships, presents supporting evidence from the included studies, and analyzes the potential explanations for the inconsistencies.

**Table 3:** Synthesis of contradictory findings in JSSE research.

Contradictory relationship	Studies supporting “positive/significant effect”	Studies supporting “No/weak effect”	Potential explanations
Effect strength of career adaptability → JSSE	1. Gerçek (2024): Career adaptability positively predicts self-perceived employability (β = 0.47, *p* < 0.01), which further predicts JSSE (β = 0.44, *p* < 0.05); the serial mediation chain reflects a strong indirect effect of career adaptability on JSSE. 2. Al-Jubari et al. (2021): Career adaptability directly and strongly predicts JSSE (β = 0.66, *p* < 0.001) among Malaysian undergraduates.	Tolentino et al. (2019): Career adaptability exerts only a weak indirect effect on JSSE via self-monitoring (Sample 1: indirect effect = 0.09, 95%CI [0.04, 0.15]; Sample 2: indirect effect = 0.12, 95%CI [0.06, 0.25]) among Thai university students.	1. Sample Heterogeneity: Gerçek’s sample (Turkish vocational students) and Al-Jubari’s sample (Malaysian undergraduates) have higher career maturity than Tolentino’s sample (Thai business students), who may have less exposure to job search practice. 2. Measurement Differences: Gerçek and Al-Jubari used full-scale career adaptability (4 dimensions: concern/control/curiosity/confidence), while Tolentino focused on career adaptability linked to self-monitoring (a narrow subdomain), weakening the observed effect.
Moderating effect of employment policy support → JSSE	Yang et al. (2022): High employment policy support (e.g., COVID-19-specific job fairs) combined with high JSSE significantly weakens the positive relationship between “perceived reduction in employment opportunities” and job search pressure (β = −0.046, *p* < 0.01) among Chinese college graduates.	DeOrtentiis et al. (2022): No significant JSSE improvement from employment policies was observed among low-social-class students; objective social class (parental income) predicts job acceptance rate (hazard ratio = 1.06, *p* = 0.02), but policy support fails to boost JSSE for disadvantaged groups.	1. Group Perception Gaps: Low-social-class students face barriers to policy access (e.g., information asymmetry, lack of institutional connections), preventing them from translating policy support into JSSE. 2. Policy Specificity: Yang’s study focused on targeted, crisis-response policies (COVID-19), while DeOrtentiis examined general policies—targeted policies are more likely to be perceived as effective, strengthening their moderating role.

The contradictions outlined in Table 3 highlight three critical lessons for JSSE research. First, sample characteristics (e.g., career maturity and social class) are not trivial; they shape how individuals perceive and respond to JSSE antecedents (e.g., career adaptability and policy support). For example, vocational students (Gerçek, 2024) may derive more JSSE from career adaptability than undergraduates (Tolentino et al., 2019) because they have more structured career preparations. Second, measurement specificity matters: using narrow subdomains of constructs (e.g., self-monitoring-linked career adaptability) versus full scales can weaken or distort the observed effects. Third, contextual factors (e.g., policy type, mentoring focus) moderate JSSE relationships—interventions such as mentoring or policy support are not “one-size-fits-all” and require alignment with individual characteristics (e.g., JSSE level) to be effective.

These contradictions underscore the need for more nuanced theoretical frameworks that incorporate heterogeneity. For instance, future JSSE models should explicitly include “moderators of antecedent effects” (e.g., social class, career maturity) to account for inconsistent findings. Practically, the contradictions suggest that interventions to boost JSSE (e.g., mentoring programs) should be tailored to individual needs—for example, low-JSSE students may need preliminary confidence-building before they can benefit from advanced mentoring.

## 4 Discussion

### 4.1 Summary of key findings

This study systematically analyzed 22 empirical studies on JSSE to map the current landscape of JSSE research, focusing on three core dimensions (antecedents, intervention mechanisms, and consequences) and identifying contradictions and biases. The key findings are as follows:

First, research on the JSSE exhibits significant imbalances and homogeneity. In terms of dimensional distribution, studies on antecedents are the most prevalent (81.8%, 18/22), focusing primarily on two categories: individual traits (e.g., career adaptability, emotional intelligence) and contextual support (e.g., mentoring, positive job search events); studies on intervention mechanisms are moderate (68.2%, 15/22), dominated by simple mediation (e.g., JSSE mediating emotional intelligence and active job search) and single moderation (e.g., extraversion moderating JSSE and job search success); studies on consequences are the scarcest (40.9%, 9/22), and nearly all (7/9) focus on short-term outcomes (e.g., job search intensity, number of job offers). In terms of research objects, 68.2% (15/22) of the studies sampled university students/graduates, while marginalized groups (e.g., refugees, ethnic minority women) and middle-aged/older job seekers accounted for only 13.6% (3/22) and 9.1% (2/22), respectively. Methodologically, cross-sectional designs dominated (77.3%, 17/22), with longitudinal (22.7%, 5/22) and experimental designs (13.6%, 3/22) being rare, limiting causal inference.

Second, core relationships in JSSE research are consistent but contain context-dependent contradictions. Consistent findings include career adaptability (especially “concern” and “confidence” dimensions) and social support positively predicting JSSE; JSSE mediating the effect of antecedents (e.g., perceived employability) on short-term job search outcomes; and individual traits (e.g., extraversion) moderating JSSE-consequence relationships. Contradictions emerge in effect strength (e.g., career adaptability → JSSE: *β* = 0.66 in Al-Jubari et al., 2021 vs. indirect effect = 0.09 in Tolentino et al., 2019) and boundary conditions (e.g., mentoring→JSSE: effective for high-JSSE individuals only in Lian et al., 2021), primarily driven by sample heterogeneity (e.g., vocational students vs. undergraduates), measurement differences (full-scale vs. subdomain constructs), and contextual variability (targeted policies vs. general policies).

### 4.2 Study implications

The findings of this study align with and extend the theoretical lineage of JSSE (from SCT to SCCT) and address gaps identified in prior meta-analyses (e.g., Kim et al., 2019), making three key contributions.

SCT emphasizes the dynamic interactions between personal, environmental, and behavioral factors. This study’s systematic classification of JSSE antecedents into “individual traits” (e.g., career adaptability, emotional intelligence) and “contextual support” (e.g., mentoring, positive events) directly operationalizes SCT’s triadic framework for the job search domain. SCCT further refines SCT into a “personal/environmental factors → self-efficacy → goals → behavior” chain; this study confirms that JSSE functions as the critical “cognitive bridge” in this chain (e.g., JSSE mediates social class → job search intensity in DeOrtentiis et al., 2022) and identifies gaps in SCCT application (e.g., lack of cross-level mechanisms linking organizational support to individual JSSE). Prior research often applied SCT/SCCT in isolation (e.g., focusing only on personal traits); this study integrated these theories to systematize JSSE’s role in vocational behavior, strengthening its theoretical grounding. Prior research often applied SCT/SCCT in isolation—even studies implicitly adopting SCCT logic, such as Lim et al. (2016) (who linked JSSE to SCCT’s career self-management model focusing on adaptive job search processes) —tended to focus only on single pathways (e.g., personal capabilities→JSSE→behavior) rather than integrating SCT’s triadic interactions with SCCT’s vocational chain. In contrast, this study integrated these theories to systematize the JSSE’s role in vocational behavior, strengthening its theoretical grounding.

Kim et al. (2019) provided a meta-analytic quantification of JSSE-variable correlations (e.g., social support-r = 0.32) but failed to address three critical gaps: (1) systematic integration of antecedents: Kim focused on bivariate correlations but did not categorize antecedents into SCCT-aligned “personal” vs. “environmental” clusters, whereas this study identifies career adaptability (individual) and mentoring (environmental) as the most impactful antecedents, clarifying their relative importance; (2) intervention mechanism specificity: Kim confirmed correlations between social support and JSSE but did not explore actionable processes (e.g., “psychosocial vs. career mentoring”), whereas this study highlights that combined psychosocial + career mentoring strengthens JSSE (Kao et al., 2021) and identifies intervention component gaps; (3) long-term consequence coverage: Kim limited consequences to short-term outcomes (e.g., interview attendance), whereas this study reveals the scarcity of research on long-term career outcomes (e.g., job retention) and non-employment spillover (e.g., mental health), aligning with SCCT’s focus on lifelong career development.

Theoretically, this study resolves the “fragmentation” of JSSE research by anchoring findings in SCT/SCCT and providing a unified framework for understanding the JSSE’s role in job searching. Methodologically, it quantifies research biases (e.g., 77.3% cross-sectional designs) and identifies causal inference limitations, thereby guiding future methodological improvements. Practically, the synthesis of contradictory findings (e.g., why mentoring fails for low-JSSE individuals) offers targeted guidance for interventions—for example, preliminary confidence-building for low-JSSE job seekers before mentoring.

### 4.3 Limitations of existing JSSE research

#### 4.3.1 Over-homogeneity in research objects and contexts

Existing JSSE research suffers from severe sample homogeneity, which limits the generalizability of the findings. Over-reliance on university students/graduates (68.2%, 15/22) means that conclusions are tailored to populations with high educational attainment, structured career support (e.g., university career services), and limited work experience. Marginalized groups, such as refugees (Morici et al., 2022), unemployed ethnic minority women (Hoye et al., 2019), and middle-aged re-entrants (Liu et al., 2021), are severely understudied (13.6%, 3/22). These groups face unique barriers (e.g., trauma for refugees and discrimination for ethnic minorities) that may reshape JSSE’s antecedents (e.g., trauma recovery as a unique antecedent) and mechanisms (e.g., ethnic networks moderating JSSE → networking behavior); however, existing models fail to account for these nuances.

Moreover, contextual homogeneity undermines external validity. Most studies focus on routine education-to-work transitions (e.g., students graduating into stable economies), with limited exploration of “special contexts” Only Yang et al. (2022) examine JSSE during COVID-19, and no study explores job search in post-conflict regions or cross-cultural migration (e.g., immigrants adapting to new labor markets). Routine contexts do not capture the uncertainty of economic recessions or cultural barriers to job searching, making the findings irrelevant for job seekers in high-volatility environments.

#### 4.3.2 Limitations in causal inference from research methods

The methodological design of the JSSE research severely limits its ability to establish causal relationships. Cross-sectional designs dominate (77.3%, 17/22), which measure variables at a single time point and cannot determine temporal order—for example, it is impossible to distinguish whether “high JSSE causes more job applications” or “more applications (and subsequent feedback) boost JSSE” (Biramo et al., 2025). Even the five longitudinal studies (22.7%) only use two waves (e.g., Guan et al., 2022: T1 = 9 months pre-graduation, T2 = 3 months pre-graduation), which fail to capture dynamic changes in JSSE over the entire job search process (e.g., JSSE fluctuations after interview rejections).

Experimental designs were rare (13.6%, 3/22), with most studies using observational data that could not rule out confounding variables. For example, the positive correlation between career adaptability and JSSE (Al-Jubari et al., 2021) may be explained by a third variable—proactive personality—which simultaneously enhances both career adaptability and JSSE. Without random assignment to intervention groups (e.g., career adaptability training vs. control), researchers cannot confirm that career adaptability causes JSSE improvements, weakening the practical value of the findings for intervention design.

#### 4.3.3 Superficial exploration of JSSE intervention mechanisms

Existing research on JSSE intervention mechanisms is overly simplistic and fails to capture the complexity of real-world job search processes. Over-focus on simple mediation and single moderation (12/15 mechanism studies) means most research explores linear, two-variable interactions (e.g., JSSE mediates emotional intelligence → job search behavior) but ignores “complex mechanisms” such as cross-level interactions (e.g., organizational job search support → team cohesion → individual JSSE) or dynamic mechanisms (e.g., JSSE changing with job search stages: early-stage vs. late-stage). For example, no study has investigated how JSSE’s role shifts from “driving application quantity” (early stage) to “sustaining persistence after rejection” (late stage), a gap that limits the understanding of JSSE’s dynamic value.

Intervention mechanisms are further obscured by vague operationalizations. Only three studies (Hamilton et al., 2019; Kao et al., 2021; Morici et al., 2022) examined JSSE-focused interventions, but none decomposed the interventions into core components. For instance, Morici et al. (2022) find that the ESPoR intervention boosts JSSE (Cohen’s d = 0.91) but does not isolate whether mock interviews, labor market training, or individual counseling drives this effect. Without component analysis, practitioners cannot replicate high-impact elements or optimize intervention dosage (e.g., 2 months vs. 4 months of training), reducing the translatability of research to practice settings.

### 4.4 Future research directions

#### 4.4.1 Broaden research objects and contexts to improve generalizability

##### 4.4.1.1 Focus on marginalized groups

Future studies should prioritize underrepresented populations to capture JSSE’s group-specific dynamics:

(1) Refugees: Build on Morici et al. (2022) to explore whether “trauma-informed career counseling” (a unique intervention for refugees) enhances JSSE more than standard counseling, and test “trauma recovery” as a unique antecedent of JSSE.
(2) Ethnic minority women: Extend Hoye et al. (2019) to compare JSSE mechanisms between minority and majority women (e.g., whether ethnic network support moderates JSSE → networking behavior more strongly for minorities).
(3) Middle-aged re-entrants: Investigate how career adaptability dimensions (e.g., “concern” about future careers vs. “confidence” in existing skills) differ in predicting JSSE compared to young graduates.

##### 4.4.1.2 Embed special contexts

Research should explore JSSE in high-volatility or cross-cultural contexts.

(1) Economic recessions: Compare JSSE antecedents pre- and post-recession (e.g., whether social support replaces career adaptability as the top predictor during recessions).
(2) Digital economy: Examine how digital skills moderate the relationship between “online job search events” (e.g., virtual interviews) and JSSE (e.g., do strong digital skills strengthen the positive effect of virtual interview feedback on JSSE?).
(3) Cross-cultural migration: Test whether cultural values (individualism vs. collectivism) moderate social support → JSSE relationships (e.g., is family support more impactful for collectivist immigrants?).

#### 4.4.2 Optimize research methods to strengthen causal inference

##### 4.4.2.1 Adopt longitudinal and experimental designs

(1) Three-wave longitudinal studies: Design studies with T1 (antecedents, e.g., career adaptability), T2 (JSSE), and T3 (consequences, e.g., job retention) to establish temporal order. For example, tracking graduates for 12 months to test whether the T2 JSSE predicts T3 career satisfaction (a long-term outcome).
(2) Randomized controlled trials: Job seekers are assigned to intervention groups (e.g., “mock interview training” vs. “resume writing training” vs. control) to isolate causal effects. For instance, test whether mock interview training (*β* = 0.35) boosts JSSE more than resume training (β = 0.18).

##### 4.4.2.2 Integrate mixed methods

Combining quantitative and qualitative approaches to capture micro-processes:

(1) Quantitative + qualitative diaries: Collect weekly JSSE surveys (quantitative) and open-ended reflections (qualitative) to explore how specific events (e.g., interview rejection, positive feedback) shape the JSSE. For example, analyze whether rejection leads to JSSE declines only when paired with low social support (qualitative theme).
(2) Interviews with practitioners: Supplement survey data with interviews with career counselors to identify unmeasured JSSE antecedents (e.g., “trust in counselor” as a contextual factor).

#### 4.4.3 Deepen exploration of JSSE mechanisms to uncover complex logic

##### 4.4.3.1 Explore complex mechanisms

(1) Multi-stage serial moderated mediation: Test models such as “cultural values → social support → JSSE → job search behavior,” with “individualism/collectivism” moderating the social support → JSSE path. For example, we verified whether social support predicted JSSE more strongly for collectivists (β = 0.42) than for individualists (β = 0.25).
(2) Cross-level mechanisms: Examine organizational-level factors (e.g., company career support programs) and team-level factors (e.g., peer job search groups) as predictors of individual JSSE. For instance, it is necessary to test whether team cohesion mediates the effect of organizational support on individual JSSE.

##### 4.4.3.2 Decompose intervention mechanisms

(1) Component analysis: Factorial designs are used to test the independent and combined effects of intervention components. For example, the effects of mock interviews (β = 0.28), resume guidance (β = 0.19), and their combination (β = 0.41) on the JSSE were compared.
(2) Dose–response models: Investigate how intervention duration/frequency affects JSSE [e.g., whether 8 weeks of counseling boosts JSSE (d = 0.85) more than 4 weeks (d = 0.42)].

#### 4.4.4 Supplement JSSE consequence research to complete its impact chain

##### 4.4.4.1 Expand consequence dimensions

(1) Long-term career outcomes: Track JSSE for 1–3 years to measure the effects on job retention (e.g., “Do high-JSSE graduates have 20% higher 1-year retention rates?”), salary growth and career advancement (e.g., promotions).
(2) Non-employment spillover effects: Measures JSSE’s impact of JSSE on mental health (e.g., job search anxiety via the Job Search Anxiety Scale) and life satisfaction (e.g., whether high JSSE reduces depression symptoms during unemployment).

##### 4.4.4.2 Clarify boundary conditions

Test moderators of “JSSE → consequence” relationships:

(1) Economic environment: Examine whether the JSSE predicts employment more strongly during recessions (e.g., β = 0.38) than during booms (β = 0.22), as a high JSSE may drive persistence when opportunities are scarce.
(2) Job type: Compare the JSSE’s effect on outcomes for manual vs. knowledge work (e.g., is the JSSE more predictive of interview success for knowledge work, where interpersonal skills matter more?).
(3) Personal resources: Explore whether social networks moderate the JSSE to job search behavior (e.g., do strong networks amplify the effect of JSSE on networking frequency?).

## 5 Conclusion

This study systematically analyzed 22 empirical studies on JSSE published between 2019 and February-2025. The study addresses three core RQs: mapping JSSE’s antecedent categories, clarifying the forms of its intervention mechanisms, and categorizing its consequence dimensions. Anchored in SCT and SCCT, this research aimed to resolve the fragmentation of existing JSSE scholarship, quantify current biases, and provide actionable guidance for theory advancement and practice. The following is a synthesis of the key conclusions, theoretical and practical implications, and a brief reflection on future priorities.

First, this study confirms that the JSSE operates as a critical cognitive hub in vocational behavior, with its antecedents, mechanisms, and consequences aligning with the core logic of SCT and SCCT, yet marked by significant imbalances and homogeneity. Regarding antecedents, individual traits (e.g., career adaptability and emotional intelligence) and contextual support (e.g., mentoring and positive job search events) emerge as the two dominant categories, directly reflecting SCT’s triadic interaction between personal and environmental factors. Career adaptability (especially the “concern” and “confidence” dimensions) and combined psychosocial + career mentoring are identified as the most impactful antecedents, addressing the gap in prior research (e.g., Lim et al., 2016; Kim et al., 2019) that focused on bivariate correlations without prioritizing key predictors. Regarding intervention mechanisms, the JSSE primarily functions as a simple mediator (e.g., linking perceived employability to job search behavior) or is moderated by individual traits (e.g., extraversion) or contextual factors (e.g., employment policy support); however, complex mechanisms (e.g., cross-level interactions, dynamic stage-dependent paths) remain largely unexamined. The consequences of the JSSE are almost exclusively short-term (e.g., job search intensity, number of job offers), with long-term career outcomes (e.g., job retention) and non-employment spillover effects (e.g., mental health) severely understudied, limiting the understanding of the JSSE’s lifelong value.

Second, the study reveals that JSSE research suffers from three critical biases that undermine its generalizability and causal validity: homogeneity in research objects (over-reliance on university students/graduates, neglect of marginalized groups such as refugees), homogeneity in contexts (focus on routine education-to-work transitions, lack of exploration in crises or cross-cultural settings), and methodological limitations (dominance of cross-sectional designs, scarcity of experiments). These biases explain the contradictory findings (e.g., varying effect sizes of career adaptability on JSSE) and highlight the need for more inclusive, rigorous research to ensure that JSSE models apply to diverse job seekers and real-world contexts.

The theoretical implications of this study lie in its integration of SCT and SCCT to systematize JSSE research. By categorizing antecedents into SCCT-aligned “personal” and “environmental” clusters, confirming JSSE’s role as a cognitive bridge in the “antecedents → behavior” chain, and identifying gaps in theory application (e.g., cross-level mechanisms), this study strengthens JSSE’s theoretical grounding and resolves the fragmentation of prior work. Practically, the findings provide targeted guidance for interventions: for university students, prioritizing career adaptability training and combined mentoring; for marginalized groups, such as refugees, integrating trauma-informed support into JSSE-building programs; and for policymakers, designing targeted employment policies (e.g., COVID-19-specific job fairs) that complement JSSE to mitigate job search pressure.

While this study synthesizes the current JSSE landscape, it has limitations: the focus on 22 studies and the exclusion of non-English literature may introduce bias. Future research should expand the literature scope and incorporate diverse cultural contexts to refine JSSE theory and practice.

In an era of global labor market volatility, the JSSE remains a vital psychological resource for job seekers navigating uncertainty. This study underscores that advancing JSSE research requires moving beyond simplistic correlations to embrace theoretical integration, methodological rigor, and inclusivity, ultimately enabling evidence-based interventions that support diverse job seekers in achieving sustainable career success.

## Data Availability

The original contributions presented in the study are included in the article/supplementary material, further inquiries can be directed to the corresponding author.
